# Revitalizing liver function in mice with liver failure through transplantation of 3D-bioprinted liver with expanded primary hepatocytes

**DOI:** 10.1126/sciadv.ado1550

**Published:** 2024-06-07

**Authors:** Bo Deng, Yue Ma, Jialyu Huang, Runbang He, Miaomiao Luo, Lina Mao, Enhua Zhang, Yuanyuan Zhao, Xiaoli Wang, Qiangsong Wang, Mingchang Pang, Yilei Mao, Huayu Yang, Lanxia Liu, Pengyu Huang

**Affiliations:** ^1^ State Key Laboratory of Advanced Medical Materials and Devices, Engineering Research Center of Pulmonary and Critical Care Medicine Technology and Device (Ministry of Education), Tianjin Key Laboratory of Biomedical Materials, Institute of Biomedical Engineering, Tianjin Institutes of Health Science, Chinese Academy of Medical Science and Peking Union Medical College, Tianjin 300192, China.; ^2^ Center for Reproductive Medicine, Jiangxi Maternal and Child Health Hospital, Jiangxi Branch of National Clinical Research Center for Obstetrics and Gynecology, Nanchang Medical College, Nanchang, China.; ^3^ Department of Liver Surgery, Peking Union Medical College Hospital, Chinese Academy of Medical Sciences and Peking Union Medical College, Beijing, China.

## Abstract

The utilization of three-dimensional (3D) bioprinting technology to create a transplantable bioartificial liver emerges as a promising remedy for the scarcity of liver donors. This study outlines our strategy for constructing a 3D-bioprinted liver, using in vitro–expanded primary hepatocytes recognized for their safety and enhanced functional robustness as hepatic cell sources for bioartificial liver construction. In addition, we have developed bioink biomaterials with mechanical and rheological properties, as well as printing capabilities, tailored for 3D bioprinting. Upon heterotopic transplantation into the mesentery of tyrosinemia or 90% hepatectomy mice, our 3D-bioprinted liver effectively restored lost liver functions, consequently extending the life span of mice afflicted with liver injuries. Notably, the inclusion of an artificial blood vessel in our 3D-bioprinted liver allowed for biomolecule exchange with host blood vessels, demonstrating, in principle, the rapid integration of the bioartificial liver into the host vascular system. This model underscores the therapeutic potential of transplantation for the treatment of liver failure diseases.

## Teaser

3D-bioprinting livers based on expandable primary hepatocytes and liver-specific bioink could save mouse liver failure.

## INTRODUCTION

The indispensable role of liver in various biochemical reactions, encompassing glycogen storage, drug metabolism, and the synthesis of secretory proteins, underscores its irreplaceable significance (*1*–*3*). Liver diseases, such as fulminant liver failure and end-stage cirrhosis, inflict irreversible damage and pose a substantial threat to patients’ lives (*4*). While orthotopic liver transplantation (OLT) remains the optimal treatment for end-stage liver disease, the escalating incidence of liver failure has created a global crisis due to a severe shortage of liver donors (*5*). The pursuit of a bioartificial liver organ, functionally and structurally similar to the native liver, stands as a paramount goal in regenerative medicine (*6*). However, current technologies fall short of replacing the native liver through OLT, leading recent efforts to focus on functional hepatocytes to compensate for lost liver tissue (*7*–*9*). Bioartificial liver support systems, using in vitro bioreactors containing hepatocytes, have sustained patients’ lives during liver failure diseases (*10*). Yet, the complexity and nonportability of these systems limit their long-term usability. Tissue engineering of hepatocytes into a transplantable bioartificial liver tissue offers a potential solution, though numerous technical challenges, including the safety and functionality of hepatocytes and the development of suitable fabrication technologies, must be addressed (*11*).

Recent advancements in three-dimensional (3D) bioprinting technologies have opened possibilities for programmable deposition of mammalian cells within bioactive hydrogels (*12*, *13*). This innovation facilitates the development of 3D biological constructs closely mimicking the complexity and heterogeneity of native tissues, providing a platform for in vitro generation of tissues and organs (*14*). In the field of bioartificial liver tissue construction, various studies have used 3D bioprinting to create functional bioartificial liver tissues suitable for drug screening or transplantation purposes (*6*). The utilization of bioactive materials during 3D bioprinting has enhanced the assembly of hepatocytes, enabling specific spatial arrangements and the development of bioartificial livers with partial functional phenotypes (*15*, *16*). This approach addresses the limitations of 2D culture and provides the mechanical microenvironment in vivo. Recent reports have demonstrated that 3D-bioprinted liver tissues can replicate partial liver-specific functions in vitro, offering a promising approach for drug screening applications (*17*, *18*). As challenges related to fabrication and functional maturation are gradually overcome, liver tissues fabricated through 3D bioprinting show tremendous potential for the future of liver transplantation.

The construction of a bioartificial liver necessitates a substantial number of functional hepatocytes. Many efforts have been undertaken to produce hepatocytes in vitro, including those derived from induced pluripotent stem cells (iPSCs) (*19*, *20*), embryonic stem (ES) cells (*21*, *22*), and the conversion of fibroblasts into hepatocytes through transdifferentiation (*23*, *24*). Nonetheless, hepatocytes obtained from these sources are associated with a time-consuming and complex procedure. Moreover, the full maturation of stem cell–derived or transdifferentiated hepatocytes remains a challenge. Recently, the chemical induction of dedifferentiation of primary hepatocytes into liver progenitor cells, capable of continuous proliferation, has opened avenues for the in vitro expansion of hepatocytes through induced dedifferentiation (*25*–*28*).

In this study, we used in vitro–expanded primary hepatocytes (eHep cells) that retain their essential hepatic functions. The eHep cells were incorporated into optimized 3D bioprinting materials to create bioartificial livers (3DP-liver). Through in vitro culturing, the 3DP-liver exhibited mature liver functional phenotypes, such as glycogen storage and drug metabolism. Upon transplantation into Fah-deficient tyrosinemia mice or 90% hepatectomized mice, the 3DP-liver rapidly established connections with the host mice through capillaries, resulting in the restoration of liver function, mitigation of liver damage, and a significant extension of the life span of the liver failure mice. In addition, we fabricated 3DP-liver containing artificial blood vessels, showcasing their ability to transport large biomolecules and glucose. This feature demonstrated the potential for direct blood vessel connection, a crucial requirement for OLT. These results underscored the potential of 3DP-liver in the treatment of liver failure diseases.

## RESULTS

### Development of 3D bioprinting liver scaffold gel

**Fig. 1.**
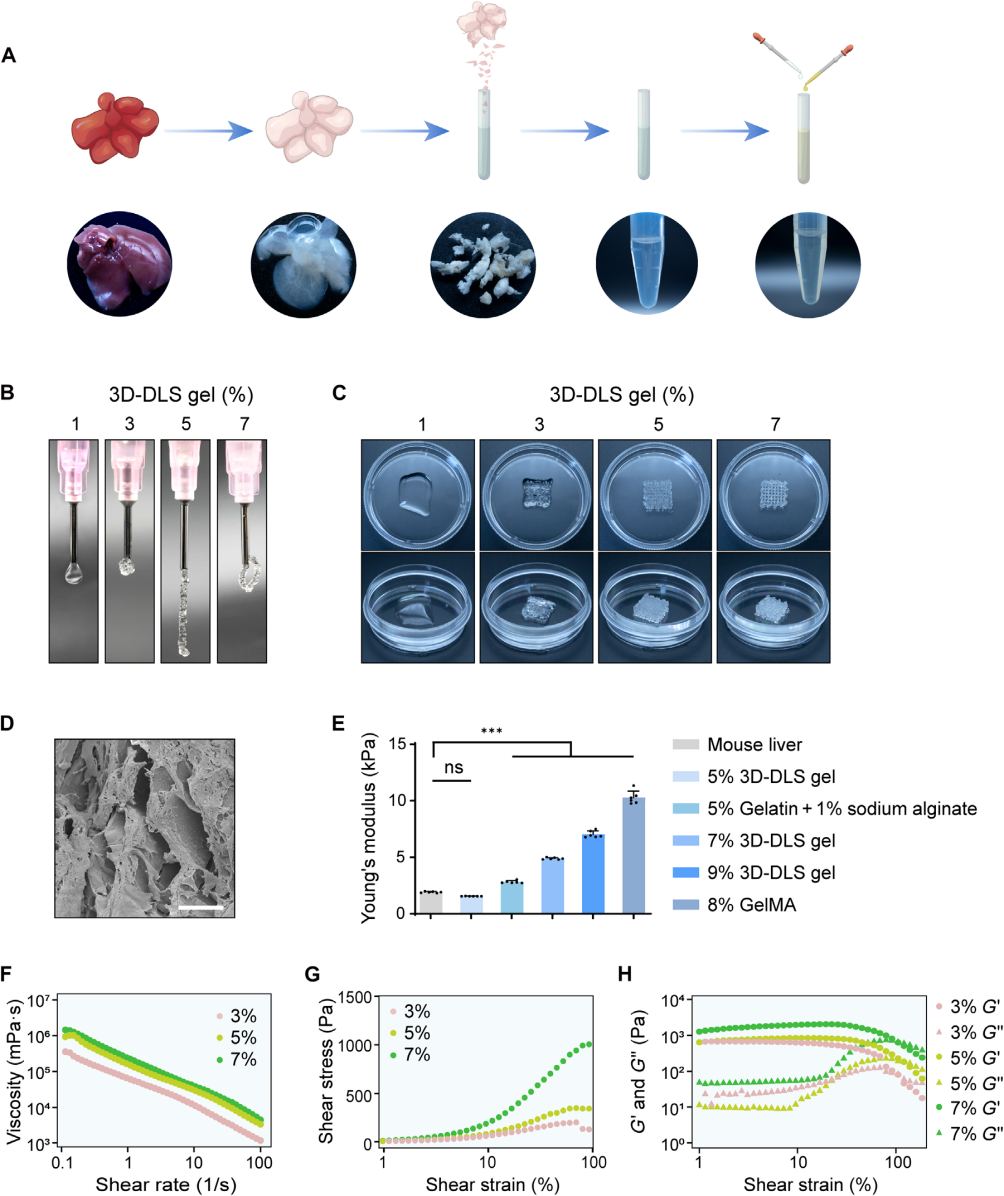
3D-DLS gel exhibits ideal mechanical and 3D printing performance. (**A**) Schematic outline of the preparation of the 3D-DLS gel. (**B**) Extrusion of the 3D-DLS gel at different concentrations (1, 3, 5, and 7%) through a 27G needle. (**C**) 3D printing of liver scaffolds with a grid-like structure using different concentrations of the 3D-DLS gel (1, 3, 5, and 7%). (**D**) SEM images of liver scaffolds printed with 5% 3D-DLS gel. Scale bar, 10 μm. (**E**) Young’s modulus of the liver scaffolds and 8-week-old C57 mouse liver printed with grids of different concentrations/materials. (**F**) Viscosity and shear-thinning behavior of the 3D-DLS gel at different concentrations (3, 5, and 7%). (**G**) Compression representative curves of the 3D-DLS gel at different concentrations (3, 5, and 7%). (**H**) Storage (*G*′) and loss (*G*″) moduli of the 3D-DLS gel as a function of the applied oscillatory strain. Premade 10% 3D-DLS comprising 10% (w/v) LDCM, 10% (w/v) gelatin, and 2% (w/v) sodium alginate, 3D-DLS with concentrations of 1, 3, 5, and 7% were prepared by diluting 10% 3D-DLS with PBS. ns, not significant. ***P < 0.001.

To fulfill the requirements for gel-based mechanical properties, 3D printing performance, and the creation of a conducive biological microenvironment for the proliferation and functional maturation of hepatocytes, we formulated a primary bioink comprising gelatin, sodium alginate, and liver decellularized matrix (LDCM). This formulation, coined as “3D bioprinting–decellularized liver scaffold gel” (3D-DLS gel), was developed. The preparation of LDCM involved a series of steps, including decellularization, freeze drying, and dissolution (Fig. 1A, detailed preparation process outlined in Materials and Methods). The results of hematoxylin and eosin (H&E) staining for LDCM are depicted in fig. S1. When utilized in isolation, the LDCM gel, with concentrations ranging from 1 to 10% at room temperature and pH = 7.0, exhibited a free-flowing consistency and a relatively soft texture, lacking the necessary structural support for the printed liver structure (fig. S2). However, through the incorporation of gelatin and sodium alginate into the LDCM, we successfully created the bioink with the required mechanical support for 3D bioprinting (Fig. 1A).

Subsequently, we evaluated the mechanical properties and 3D printing performance of the 3D-DLS gel across various concentrations. A premade gel of 10% 3D-DLS, consisting of 10% (w/v) LDCM, 10% (w/v) gelatin, and 2% (w/v) sodium alginate, was used as a base. Subsequently, 3D-DLS with concentrations of 1, 3, 5, and 7% were prepared by diluting the initial 10% 3D-DLS solution with phosphate-buffered saline (PBS). Extruding a 5% 3D-DLS gel concentration through a 27G needle resulted in a smooth and continuous filament, contrasting with other 3D-DLS gel concentrations that did not flow smoothly during the bioprinting process (Fig. 1B). In addition, the 5% 3D-DLS gel closely resembled a solid hydrogel and displayed excellent shape retention under static conditions (Fig. 1C).

Scanning electron microscopy (SEM) analysis of the cross-sectional microstructure of the printed constructs using 5% 3D-DLS gel revealed a complex and densely porous structure, providing ample space for hepatocyte proliferation and facilitating their liver functions (Fig. 1D). Moreover, the Young’s modulus of the constructs using 5% 3D-DLS gel closely mirrored that of mouse liver tissue, offering a mechanical microenvironment akin to natural liver tissue for the host and maturation of hepatocytes (Fig. 1E). In contrast, the gelatin methacryloyl (GelMA) hydrogel, previously used in the construction of 3D-bioprinted liver tissue, exhibited a significantly higher Young’s modulus (Fig. 1E) (*29*).

To quantify the rheological behavior of 3D-DLS gel, we measured viscosity at different concentrations. The flow properties of bioinks were investigated by measuring shear viscosity as a function of shear rates. As indicated in Fig. 1F, the viscosity of the bioink decreased with shear rate, demonstrating non-Newtonian shear-thinning viscosity behavior, which is essential for the printing process. In addition, viscosity increased with increasing bioink concentration at a given shear rate. This shear-thinning behavior suggests that the bioink maintains low viscosity at high shear rates, reducing the risk of cellular damage during the bio printing process.

The mechanical properties of bioink with different concentrations were also studied, and the shear strain–stress curves are shown in Fig. 1G. The bioink demonstrated good elasticity, with a failure strain exceeding 90% for all concentrations. Last, in Fig. 1H, during the strain increase from 1 to 25%, both *G*′ and *G*″ (representing elastic and viscous components) remained relatively stable. However, as the strain continued to increase beyond 25%, both *G*′ and *G*″ sharply decreased and crossed over after 100%, suggesting the disruption of the hydrogel network.

### In vitro expansion of primary mouse hepatocytes

**Fig. 2.**
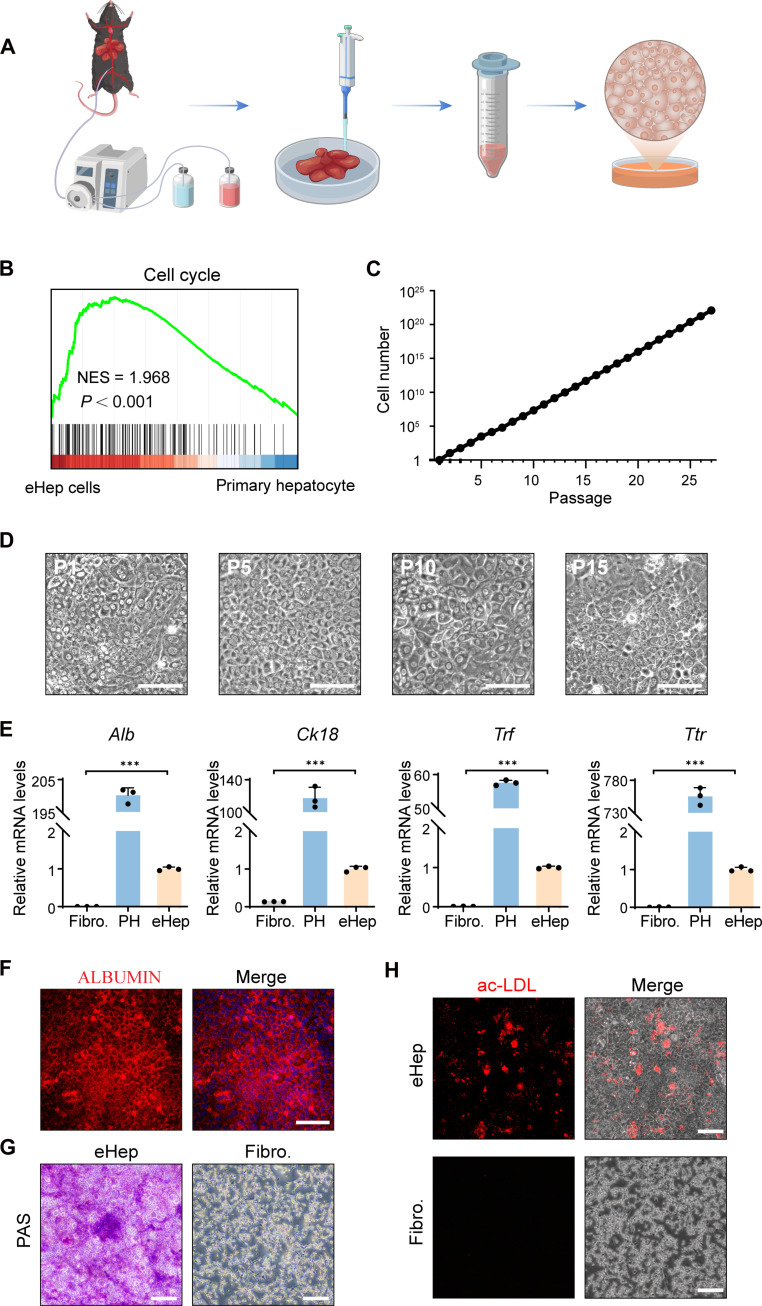
In vitro cultivation using HEM culture medium enables the expansion of mice hepatocytes with both proliferative capacity and mature liver functionality. (**A**) Isolation of primary mice hepatocytes achieved through the perfusion method. (**B**) Images of cultured eHep cells at 15 passages in the HEM. Scale bar, 100 μm. NES, normalized enrichment score. (**C**) Growth curves of cultured eHep cells under the HEM. (**D**) Transcriptomic analyses of cell cycle–related genes. Freshly isolated mice hepatocytes and eHep cells were used for mRNA microarray analyses. (**E**) Expression of liver-related genes in mice fibroblasts (Fibro.), freshly isolated mice primary hepatocytes (PH), and eHep cells (eHep, P10) measured by quantitative polymerase chain reaction (qPCR). (**F**) Expression of ALBUMIN in eHep cells determined by immunofluorescent staining. The percentages of ALBUMIN-positive cells were quantified. Scale bar, 100 μm. (**G**) Glycogen storage in eHep cells at P10 analyzed by periodic acid–Schiff (PAS) staining. Scale bar, 200 μm. (**H**) Intake of ac-LDL in eHep cells (red staining). Scale bar, 200 mm. ***P < 0.001.

To establish an eHep cell culture, the hepatocytes were isolated from mouse livers and induced to proliferate using hepatocyte expansion medium (HEM) (Fig. 2A). The eHep cells demonstrated the ability for continuous passaging while maintaining their characteristic polygonal epithelial morphology (Fig. 2, B and C). Notably, our study demonstrated that primary mouse hepatocytes could be passaged for more than 25 generations, reaching a theoretical cell count exceeding 1 × 10^20^ (Fig. 2C). Gene set enrichment analysis (GSEA) also revealed an enrichment of the cell cycle gene set in eHep cells (Fig. 2D). eHep cells sustained the expression of hepatocyte-enriched genes, such as *Alb*, *Ck18*, *Ttr*, and *Trf*, albeit at reduced levels. Furthermore, 96.9 ± 1.5% of the analyzed eHep cells were positively stained by the hepatic marker protein ALBUMIN (Fig. 2F). In addition, we assessed the liver functions of eHep cells, demonstrating their capacity to store glycogen (Fig. 2G) and uptake acetylated low-density lipoprotein (ac-LDL; Fig. 2H). These results suggest that eHep cells effectively maintain hepatic gene expression and function.

### Biocompatibility assessment of 3DP-liver

**Fig. 3.**
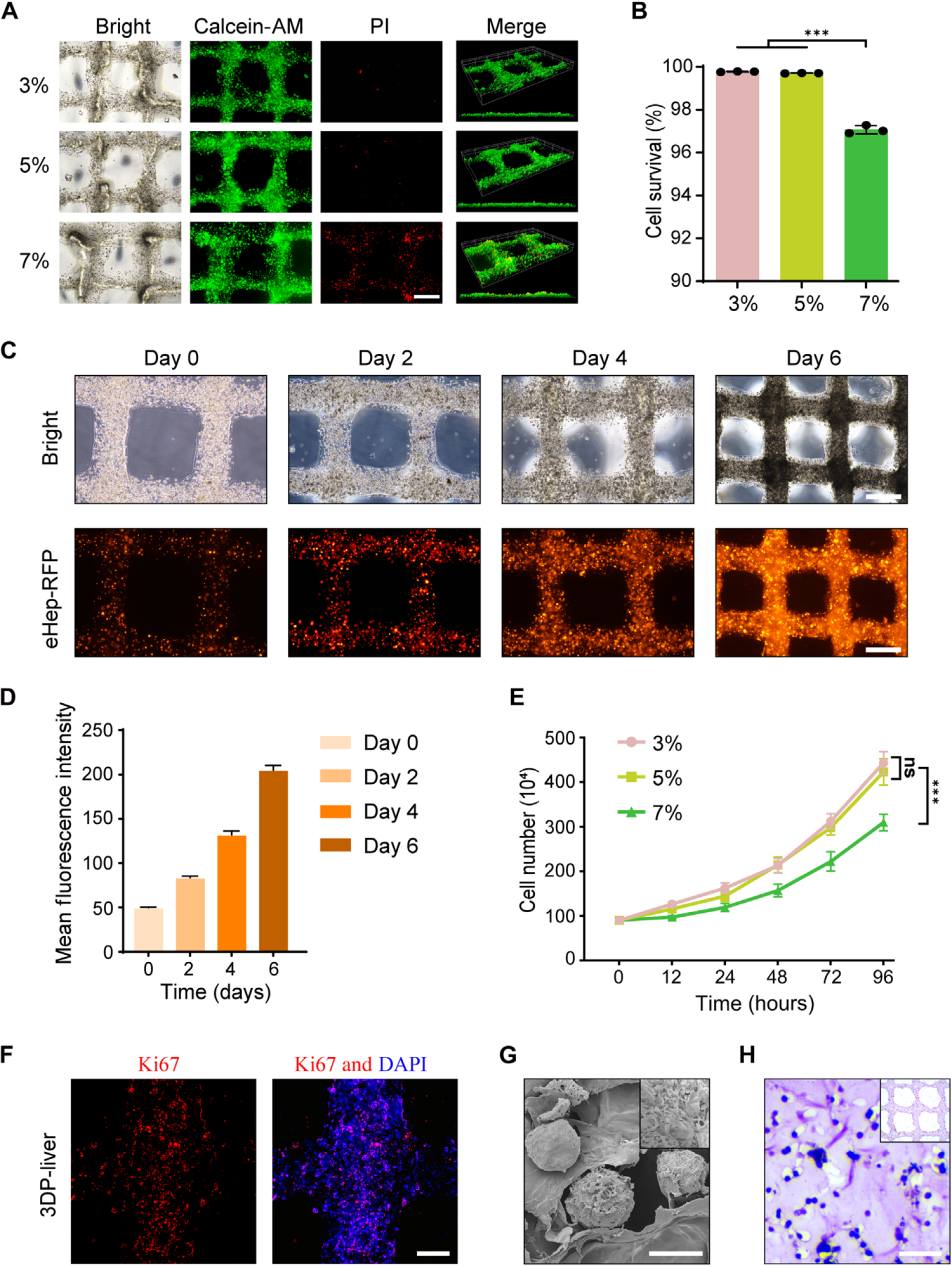
3D-DLS gel demonstrates exceptional biocompatibility and facilitates the sustained proliferation of eHep cells. (**A**) Representative live-dead staining images of 3DP-liver structures at day 5 using different 3D-DLS concentrations. Live and dead cells were labelled with calcein-AM (green) and PI (red), respectively. Scale bar, 500 μm. (**B**) Assessment of eHep cells viability within the printed structures on day 5. Different concentrations of the 3D-DLS gel were used. (**C**) Representative optical images of 3DP-liver (using 5% 3D-DLS gel) at days 0, 2, 4, and 6. For better visualization, eHep-RFP cells were used. Scale bar, 5 mm. (**D**) Mean fluorescence intensity of eHep-RFP cells in 3DP-liver (using 5% 3D-DLS gel) at days 0, 2, 4, and 6. (**E**) Growth curves of eHep-RFP cells analyzed at different concentrations of 3D-DLS gel (3, 5, and 7%). (**F**) Proliferation of eHep cells assessed by Ki67 immunofluorescent staining on day 5. The percentages of Ki67-positive cells were quantified. Scale bar, 200 μm. DAPI, 4′,6-diamidino-2-phenylindole. (**G**) SEM images of 3DP-liver with 5% 3D-DLS gel. Scale bar, 15 μm (**H**) H&E staining of formalin-fixed paraffin-embedded 3DP-liver sections. Scale bar, 50 μm. ***P < 0.001.

Subsequently, we evaluated the biocompatibility of the 3D-DLS gel for eHep cells during 3D bioprinting. We dispersed eHep cells at a concentration of 5 × 10^6^ cells/ml in various concentrations of 3D-DLS gel for 3D bioprinting. The resulting 3DP-liver tissue was cultured in HEM for 5 days. Staining of 3DP-liver with calcein acetoxymethyl ester (AM) and propidium iodide (PI) revealed a remarkable cell survival rate of more than 99% with 3 and 5% 3D-DLS gel concentrations. However, a significant increase in cell death was observed at a higher 3D-DLS gel concentration (7%), emphasizing the importance of 3D-DLS gel concentration in supporting the long-term survival of eHep cells (Fig. 3, A and B).

To investigate the potential of 3D-DLS gel in creating a biocompatible microenvironment conducive to sustained eHep cell proliferation, we enhanced the visibility of eHep cells by overexpressing red fluorescent protein (RFP) using lentiviral transduction (eHep-RFP cells). Following the in vitro culture of 3DP-liver constructed with eHep-RFP cells, a consistent rise in RFP fluorescence was observed, signifying the ongoing proliferation of eHep-RFP cells (Fig. 3, C to E). Notably, 3 and 5% concentrations of 3D-DLS gel were found to better support the proliferation of eHep-RFP cells compared to the 7% concentration. Simultaneously, incorporating the decellularized liver matrix has resulted in a modest enhancement in the proliferation rate of eHep cells compared to using gelatin/sodium alginate gel alone, though the improvement is not markedly significant (fig. S3). Nevertheless, after 5 days of culture with 5% 3D-DLS gel, 23.3 ± 2.4% of eHep cells were positively stained with Ki67, demonstrating robust cell proliferation in the in vitro–cultured 3DP-liver (Fig. 3F). Morphological examination of the 3DP-liver after 5 days revealed that the majority of eHep cells grew in clusters within the porous structure (Fig. 3, G and H). These findings suggest that the 5% 3D-DLS gel exhibits excellent printing performance and biocompatibility, leading us to select it for subsequent experiments.

### In vitro liver functions of 3DP-liver

**Fig. 4.**
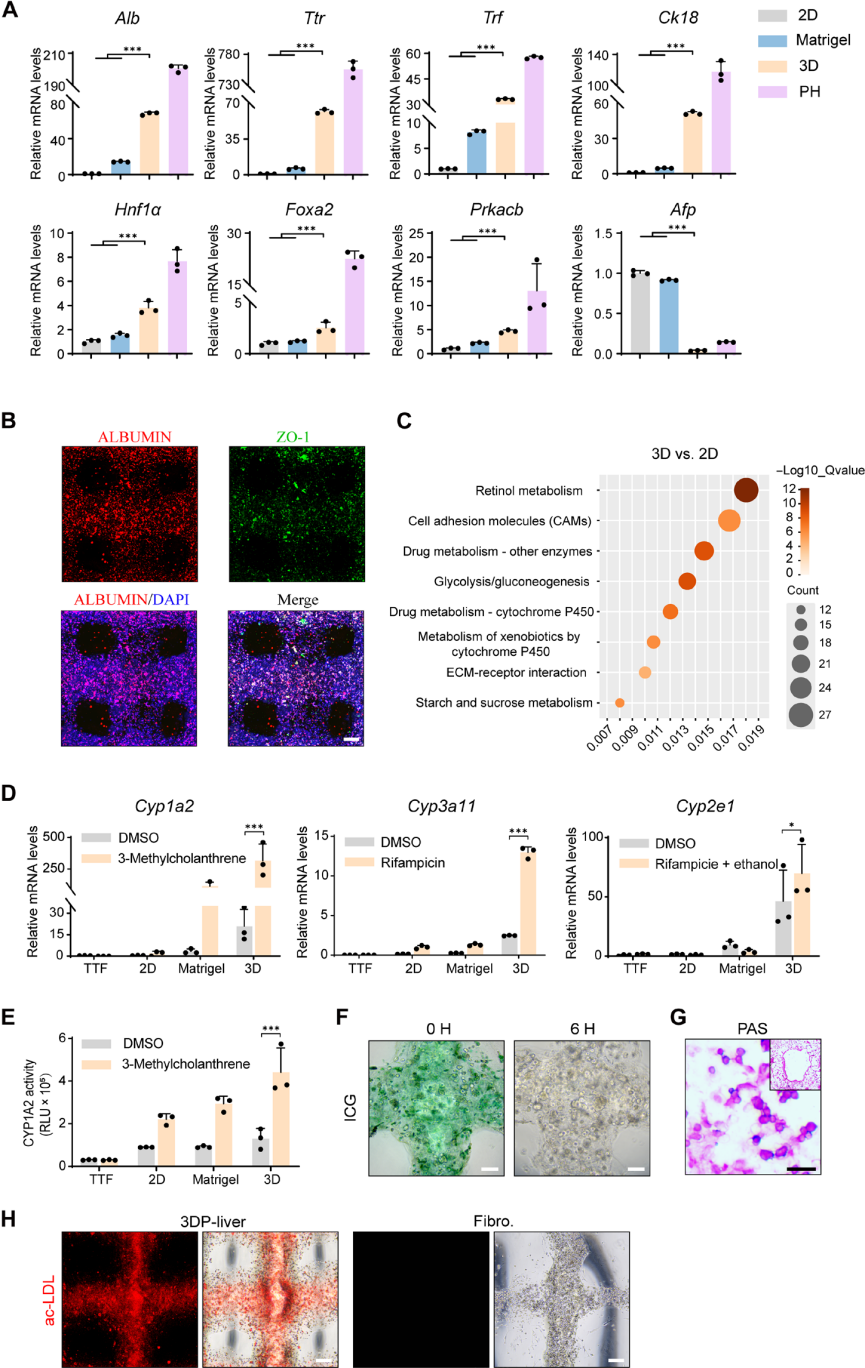
3DP-liver exhibit liver functions in vitro. (**A**) qPCR analysis of the expression of the hepatic marker gene expression in 2D-culture (2D), Matrigel-based 3D culture (Matrigel), freshly isolated mice primary hepatocytes (PH), and 3DP-liver (3D) after 5 days of in vitro culturing. (**B**) Expression of ALBUMIN and Zo1 in 3D-livers determined by coimmunofluorescent staining. Scale bar, 200 μm. (**C**) KEGG pathway analyses of the 933 genes that were specifically up-regulated in 3DP-liver. (**D**) mRNA levels of uninduced CYP and induced CYP enzymes determined by qPCR. Tail-tip fibroblasts (TTF); 2D-cultured (2D); Matrigel-based 3D culture (matrigel); 3DP-liver (3D); *Cyp1a2* was induced by 3-methylcholanthrene. *Cyp3a11* was induced by rifampicin. *Cyp2e1* was induced by rifampicin and ethyl alcohol. DMSO, dimethyl sulfoxide. (**E**) P450-Glo assay of cytochrome activity (CYP1A2) in 3DP-liver after 5 days of in vitro culturing. (**F**) ICG uptake and release in 3DP-liver (green). Scale bar, 100 μm. (**G**) Glycogen storage determined by PAS staining. Scale bar, 50 μm. (**H**) Intake of ac-LDL (red staining) in 3DP-liverand print containing TTF (Fibro.). Scale bar, 200 μm. *P < 0.05 and ***P < 0.001.

Given that the 3DP-liver comprised in vitro–expanded primary hepatocytes, we aimed to evaluate its liver functions. Consistent with prior studies, the 3DP-liver exhibited elevated expression levels of hepatic functional genes, including *Alb*, *Ttr*, *Trf*, and *Ck18*, when compared to 2D-cultured eHep cells. Notably, the expression of hepatic functional genes in the 3DP-liver surpassed those in eHep cell–derived liver organoids embedded in Matrigel, suggesting that the 3D-DLS gel provides a microenvironment conducive to hepatocyte maturation compared to mice primary hepatocytes, 2D culture, gelatin/sodium alginate–based 3D culture, and Matrigel-based 3D culture (Fig. 4A and fig. S4). The expression of liver-enriched transcription factors, including *Hnf1*α and *Foxa2*, which are critical for hepatocyte cell fate, was significantly induced in the 3DP-liver, indicating enhanced hepatic cell fate determination of eHep cells within this environment (*23*). *Prkaca*, *Prkacb*, and *Prkx*, the adenosine 3′,5′-monophosphate (cAMP)–dependent kinases known for their significant role in hepatocyte maturation, were also notably up-regulated in the 3DP-liver (figs. S5 and S6) (*30*). In addition, the 3DP-liver showed a marked reduction in the expression of the fetal liver marker *Afp*, further substantiating the maturation of eHep cells within 3DP-liver (Fig. 4A). Immunofluorescence staining confirmed the expression of ALBUMIN and zonula occludens-1 (Fig. 4B), indicating further maturation of eHep cells within 3DP-liver.

To comprehensively explore the impact of the 3DP-liver microenvironment on eHep cells, we conducted a comparative analysis of the global expression profiles between eHep cells cultured in 2D and those within 3DP-liver. Differences in the global expression profiles of eHep cells between 3DP-liver and 2D cultures were observed (fig. S7). Within 3DP-liver, eHep cells displayed 933 up-regulated genes and 1590 down-regulated genes (figs. S8 and S9). Subsequent Kyoto Encyclopedia of Genes and Genomes (KEGG) analyses revealed an augmentation in liver functional pathways within the 3DP-liver, including retinol metabolism, drug/xenobiotics metabolism, and glycolysis/gluconeogenesis. Notably, pathways related to cell adhesion and extracellular matrix (ECM)–receptor interaction, crucial for the formation of 3D tissue structures, were significantly enriched (Fig. 4C). The GSEA further demonstrated that genes related to metabolism were significantly enriched in both 3DP-liver and primary mouse hepatocytes, reflecting the heightened metabolic activity of eHep cells in the 3DP-liver (fig. S10).

The liver, serving as the primary organ for drug and xenobiotics metabolism, exhibited higher expression levels of genes encoding detoxification enzymes, particularly cytochrome P450 enzymes in 3DP-liver, as indicated by KEGG analysis and GSEA (Fig. 4C and fig. S10). An integral function of the liver is to respond to drugs or xenobiotics by up-regulating the expression of *Cyp450* genes. In this study, we treated the 3DP-liver with *Cyp450* inducers, including 3-methylcholanthrene, rifampicin, ethyl alcohol, and barbituric acid, respectively. Notably, the expression of *Cyp1a2* (responsive to 3-methylcholanthrene), *Cyp3a11* (responsive to rifampicin), *Cyp2e1* (responsive to rifampicin and ethyl alcohol), *Cyp3a41b* (responsive to 3-methylcholanthrene), and *Cyp1a1* (responsive to rifampicin and barbituric acid) was significantly induced in 3DP-liver (Fig. 4D and figs. S11 and S12). The 3DP-liver also acquired CYP1A2 activity to metabolize the derivative of (4S)-4,5-dihydro-2-(6′-hydroxy-2’benzothiazolyl)-4-thiazolecarboxylic acid, further inducible by its inducer 3-methylcholanthrene (Fig. 4E).

In addition to *Cyp450* enzyme activities, the 3DP-liver exhibited many important liver functions, including absorption/release of indocyanine green (ICG) (Fig. 4F), glycogen storage (Fig. 4G), and uptake of ac-LDL (Fig. 4H). These results demonstrated that 3DP-liver acquired numerous mature liver functions in vitro.

Furthermore, aside from the previously mentioned changes in genes related to liver function, alterations in signaling pathways associated with cell growth, such as the cell adhesion molecule pathway and ECM-receptor pathway, were observed (figs. S13 and S14). Several genes encoding ECM proteins (*Col6a2*, *Tnc*, *Tnxb*, *Reln*, etc.) and genes encoding adhesion proteins (*Lama3*, *Lamb1*, *Lamb3*, *Tnxb*, *Ncam1*, *Cldn*, etc.) showed an increase in expression. These changes can likely be attributed to the presence of liver cell–derived ECM components in the printing ink and the 3D culture environment.

### Therapeutic potential of 3DP-liver in mice with liver failure

To assess the in vivo safety and therapeutic potential of 3DP-liver, we conducted a transplantation study in C57BL/6NCrl mice. Notably, the structural integrity of 3DP-liver remained intact 7 days and 1 month after transplantation (fig. S15). In vitro culture experiments demonstrated that 3DP-liver effectively preserved cell loading for a minimum of 4 days, indicating minimal cell escape from the 3DP-liver (fig. S16). The transplantation of 3DP-liver exhibited no adverse effects on mouse survival, as illustrated in fig. S17. In addition, an analysis of blood indices after 7 days after transplantation revealed minimal to no impact on these parameters, further affirming the in vivo safety of 3DP-liver (fig. S18).

**Fig. 5.**
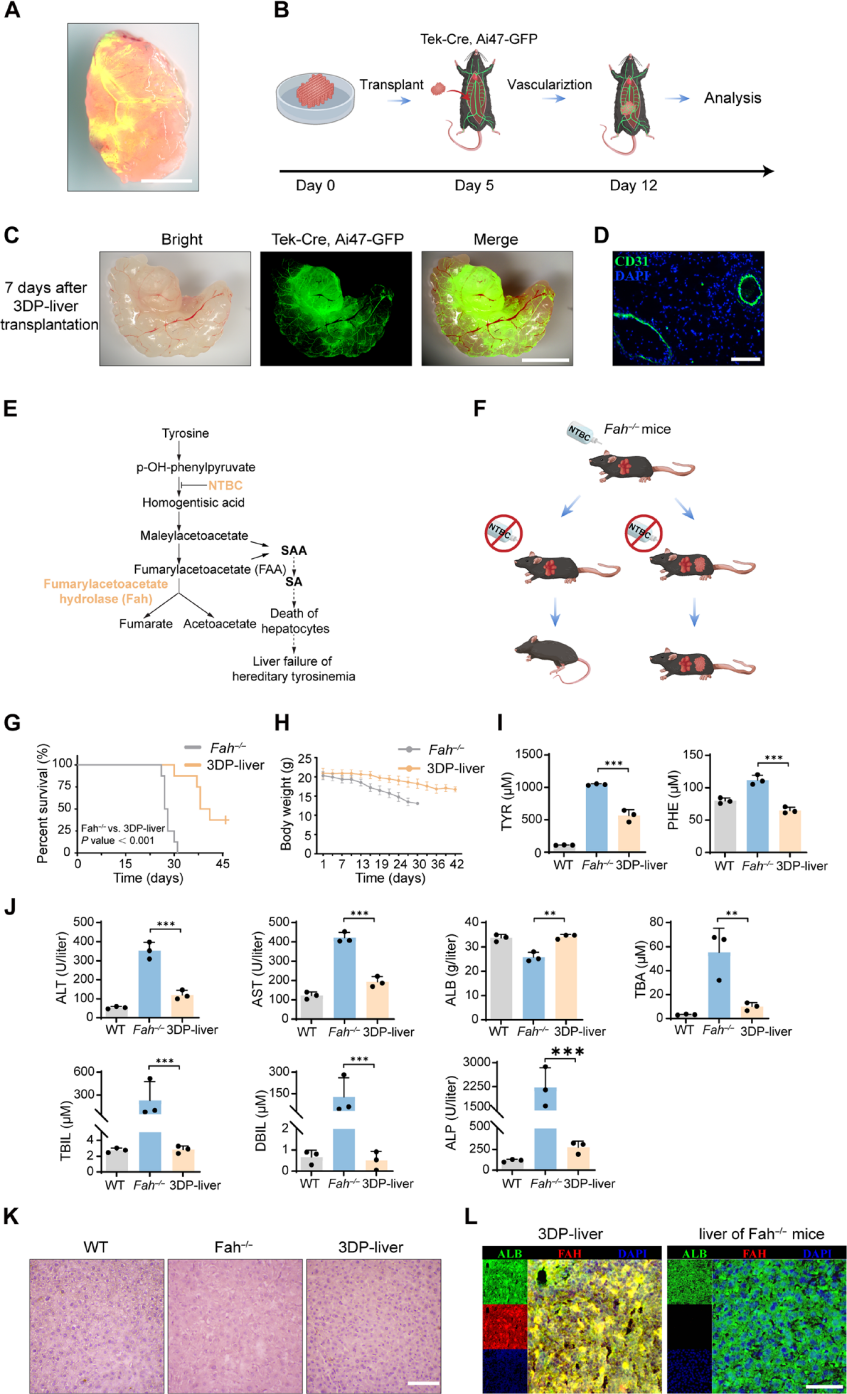
3DP-liver transplantation rescues liver failure mice. (**A**) Macroscopic observation of the 3DP-liver transplantation after 1 week. Intravenous injection of FID reveals the vascular system after 10 min. Red, eHep-RFP cells; green, FID. Scale bar, 5 mm. (**B**) Schematic outline of 3DP-liver transplanted into the mesentery of Tek-Cre, Ai47-GFP mice. (**C**) Vascularization of 3DP-liver transplanted into the mesentery of Tek-Cre, Ai47-GFP mice after 7 days. Scale bar, 5 mm. (**D**) Immunofluorescence staining of CD31 in 3DP-livers 7 days after transplantation into C57 mice. Scale bar, 100 μm. (**E**) Schematic outline of the pathogenesis of *Fah^−/−^* mice. SA, succinylacetone; SAA, succinylacetoacetate. (**F**) Schematic outline of 3DP-liver transplanted into the mesentery of *Fah^−/−^* mice. (**G**) Survival curve of *Fah^−/−^* mice with 3DP-liver transplanted (3DP-liver, *n* = 8) and control *Fah^−/−^* mice (*Fah^−/−^*, *n* = 8) after NTBC withdrawal. (**H**) Body weight of *Fah^−/−^* mice (*n* = 8) and control *Fah^−/−^* mice (*n* = 8) measured after NTBC withdrawal. (**I**) Serum levels of TYR (tyrosine) and PHE (phenylalanine) in wild-type (WT, *n* = 3), *Fah^−/−^* mice (*Fah^−/−^*, *n* = 3), and *Fah^−/−^* mice with 3DP-liver transplanted (3DP-liver, *n* = 3, sera collected at 3 weeks after transplantation). (**J**) Serum levels of ALT, AST, ALB, TBA, TBIL, DBIL, and ALP in WT (*n* = 3), *Fah^−/−^* mice (*n* = 3), and *Fah^−/−^* mice with 3DP-liver transplanted (*n* = 3, sera collected at 3 weeks after transplantation). (**K**) H&E staining of liver from WT, *Fah^−/−^* mice, and *Fah^−/−^* mice with 3DP-liver transplanted. Scale bar, 100 μm. (**L**) Immunofluorescence costaining of ALBUMIN and FAH in 3DP-livers 1 month after transplantation into C57 mice and *Fah^−/−^* mice. Scale bar, 50 μm. **P < 0.01 and ***P < 0.001.

Vascularization is crucial for maintaining the viability of 3D-bioprinted tissues in vivo. By injecting fluorescein isothiocyanate dextran (FID) into the tail vein of mice with transplanted 3DP-liver, we observed that FID could penetrate the interior of the 3DP-liver via newly formed capillaries (Fig. 5A). To further examine the development of new vascular systems in the 3DP-liver after transplantation, we used the Tek-Cre, Ai47–green fluorescent protein (GFP) mouse model, which labels endothelial cells with GFP under the control of the endothelial-specific gene promoter *Tek* (also known as *Tie2*) (Fig. 5B). Neovascularization was evident around the 3DP-liver as early as 1 day after transplantation, with the capillary density around the 3DP-liver increasing progressively over time (figs. S19 and S20). The hardness of the 3DP-liver influences the vascularization process. Using a 7% 3D-DLS gel slowed the rate of vascularization, with minimal neovascular formation observed by the third day (fig. S21). Notably, a functional GFP^+^ vascular network was present throughout the entire 3DP-liver 7 days after transplantation (Fig. 5C). Seven days after transplantation into C57 mice, immunofluorescence staining demonstrated the presence of newly formed blood vessels within the 3DP-liver (Fig. 5D).

Over time, following the transplantation, there was a significant increase in fibronectin expression, indicative of substantial ECM remodeling (fig. S22). After transplantation, eHep cells exhibited slow proliferation, reaching a peak around the seventh day (fig. S23). One month after transplantation, immunofluorescence staining showed that Ki67-positive cells constituted less than 0.1% of the total, suggesting that eHep cell proliferation had ceased. This finding underscores the excellent safety profile of the 3DP-liver (fig. S24).

To explore the therapeutic potential of 3DP-liver, we transplanted them into a Fah-deficient hereditary tyrosinemia mouse model. *Fah* encodes the tyrosine metabolism enzyme fumarylacetoacetate hydrolase. A deficiency in this enzyme leads to the accumulation of succinylacetone and succinylacetoacetate in hepatocytes, causing hepatic cell death and liver failure, a condition known as hereditary tyrosinemia (Fig. 5E). In this study, *Fah^−/−^* mice, maintained with nitisinone (NTBC) in their drinking water, received a 3DP-liver transplantation into the mesentery (Fig. 5F). NTBC was immediately removed upon transplantation to induce tyrosinemia and liver injury. Typically, *Fah^−/−^* mice experience gradual weight loss and succumb to liver failure around 20 to 30 days after NTBC removal (Fig. 5G). *Fah^−/−^* mice receiving 3DP-liver transplantation maintained their body weights and exhibited a significantly enhanced survival rate and prolonged life span, suggesting that 3DP-liver transplantation compensated for the lost functions of the native liver during tyrosinemia-induced liver injury (Fig. 5H).

Consistent with prior studies, Fah deficiency–induced tyrosine metabolism disorder resulted in abnormally elevated serum concentrations of tyrosine and its precursor, phenylalanine (Fig. 5I). Notably, 3DP-liver transplantation significantly reduced the serum concentrations of tyrosine and phenylalanine (Fig. 5I). Liver injury also impairs overall amino acid metabolism, as evidenced by significantly increased serum amino acid levels, which were rescued by 3DP-liver transplantation (fig. S25). These findings suggest that the eHep cells within the 3DP-liver compensated for the tyrosine metabolism disorder, highlighting the therapeutic potential of 3DP-liver.

Furthermore, 3DP-liver transplantation markedly reduced injury to the native liver and enhanced overall liver functions. This improvement was evident through a reduction in serum levels of alanine aminotransferase (ALT), aspartate aminotransferase (AST), total bile acid (TBA), total bilirubin (TBIL), direct bilirubin (DBIL), and alkaline phosphatase (ALP), accompanied by an increase in serum albumin (ALB) levels after 7 days of transplantation (Fig. 5J). The amelioration of liver injury with 3DP-liver transplantation was further supported by H&E staining (Fig. 5K) and representative images (fig. S26). One month following 3DP-liver transplantation into *Fah^−/−^* mice, the eHep cells in 3DP-liver matures functionally, expressing mature liver-specific proteins, such as ALBUMIN and FAH (Fig. 5L). In addition, C57BL/6NCrl mice subjected to 3DP-liver transplantation demonstrated a significantly enhanced life span following 90% hepatectomy, providing robust evidence for the therapeutic potential of this approach (figs. S27 and 28).

### Transplantation of 3DP-liver with artificial blood vessels

**Fig. 6.**
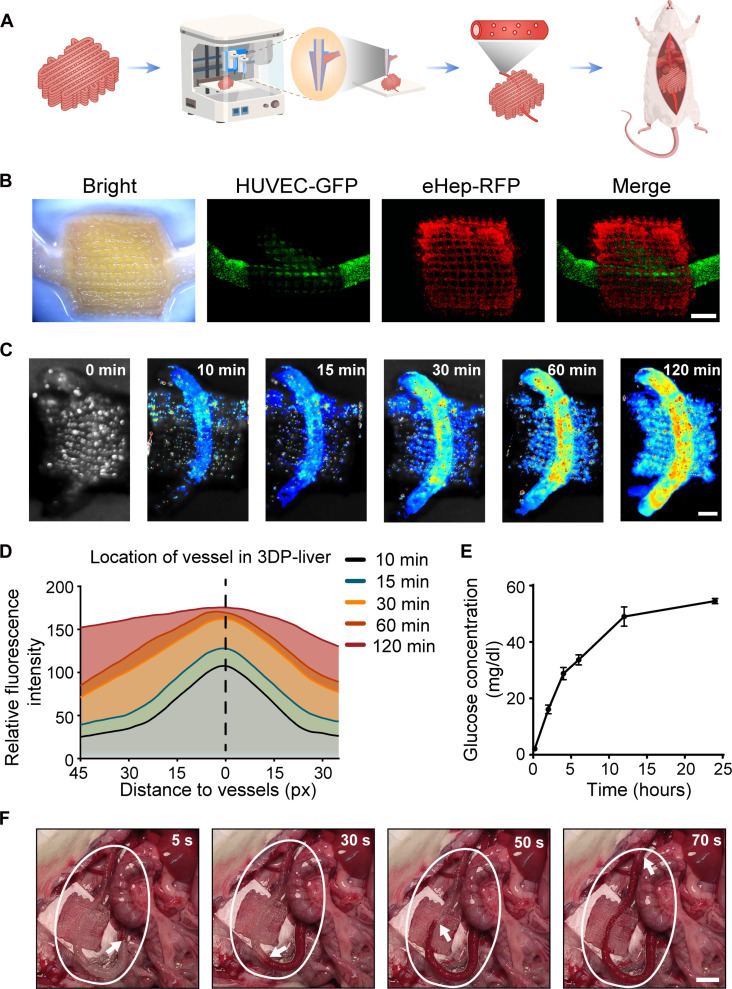
Transplantation of 3DP-liver with artificial blood vessels. (**A**) Schematic outline of the preparation of 3DP-liver with artificial blood vessels. (**B**) Representative optical images of 3DP-liver with artificial blood vessels. For better visualization, eHep-RFP cells and HUVEC-GFP were used. Scale bar, 5 mm. (**C**) Diffusion of Cy7 fluorescent dye within 3DP-liver with artificial blood vessels. Scale bar, 5 mm. (**D**) Mean fluorescence intensity at various time points in different regions of the 3DP-liver after perfusion with Cy7 fluorescent dye. (**E**) Glucose concentration in PBS after perfusion with glucose via artificial blood vessels. (**F**) Surgical anastomosis of the 3DP-liver with artificial blood vessels in rat was performed through the inferior vena cava and portal vein. Blood perfusion was established immediately after anastomosis. Scale bar, 1 cm.

The exploration of neovascularization in 3DP-liver led us to investigate connecting them to native vascular systems through the direct integration of artificial blood vessels. Using a layer-by-layer printing approach, we initially printed a 3DP-liver layer that was 5 mm thick and subsequently integrated an artificial blood vessel through coaxial printing. The construction of the artificial blood vessel involved suspending human umbilical vein endothelial cells (HUVECs) (final concentration 5 × 10^6^/ml) in a premade bioink mixture comprising 8% GelMA, 1% sodium alginate, 0.25% Lithium Phenyl(2,4,6-trimethylbenzoyl)phosphinate (LAP), and 1.6% polyethylene oxide (PEO). The addition of PEO induced the formation of scattered micropores along the artificial blood vessels, the diameter of the sacrificial material-formed nanopores measuring around 10 to 20 μm (fig. S29), which facilitates molecular exchange between the blood and 3DP-liver. This coaxially printed blood vessel was then covered by another layer of 5-mm-thick 3DP-liver (Fig. 6A). To enhance the visualization of the 3DP-liver containing an artificial blood vessel, we used eHep-RFP and HUVECs expressing GFP (HUVEC-GFP) in the construction of the 3DP-liver (Fig. 6B).

To examine the diffusion of biomolecules within the 3DP-liver, we dissolved the Cy7 fluorescent dye in PBS and subjected it to in vitro perfusion. Over the extended perfusion period, Cy7 gradually diffused uniformly throughout the 3DP-liver within 120 min, with the artificial blood vessels serving as the central distribution pathway (Fig. 6, C and D). Furthermore, a steady diffusion of glucose from the artificial blood vessel was observed when we perfused the blood vessel with glucose (100 mg/dl), positioning the artificial blood vessels within a 6-cm dish filled with PBS (Fig. 6E). These results demonstrate that the artificial blood vessel in the 3DP-liver is capable of gradual diffusion of biomolecules.

Subsequently, we connected the 3DP-liver to the inferior vena cava and portal vein of a rat to perform an OLT. The artificial blood vessel within the 3DP-liver allowed blood circulation throughout the body (Fig. 6F and movie S1). No instances of blood leakage from the artificial blood vessel were observed (movie S2). Thus, the 3DP-liver, containing artificial blood vessels, can be transplanted into rats through a direct blood connection, demonstrating the potential for in situ liver transplantation.

## DISCUSSION

The creation of a transplantable bioartificial liver holds immense potential in the field of regenerative medicine. In this study, we used 3D bioprinting technology to fabricate 3DP-liver, demonstrating liver functions both in vitro and in vivo. Significantly, the transplantation of 3DP-liver effectively compensated for lost liver functions in the context of liver injuries. Notably, this transplantation notably enhanced the life span of Fah-deficient tyrosinemia mice. Therefore, this proof-of-principle work underscores the therapeutic potential of 3DP-liver.

The availability and functionality of seed cells play pivotal roles in determining the functions and in vivo behavior of 3DP-liver. Commonly used hepatic cell lines, such as HepG2 cell lines, derived from hepatocellular carcinomas, often experience a loss of mature liver functions (*31*, *32*).

This limitation renders them unsuitable for constructing bioartificial livers for therapeutic purposes, primarily because of safety concerns. In contrast, hepatocytes derived from pluripotent stem cells, including iPSCs and ES cells, showcase diverse liver functions and have garnered significant attention for their potential as hepatic cell sources for the construction of 3DP-liver. Nevertheless, the high costs, relatively complex, and time-consuming processes required to generate functional hepatocytes from iPSCs or ES cells remain substantial challenges (*33*). Recent breakthroughs in the in vitro expansion of primary hepatocytes have provided a promising solution. In vitro–expanded hepatocytes offer an easily preparable process while preserving essential liver functions, making them a promising hepatic cell source for fabricating large-scale, cost-effective bioartificial livers with improved liver functions and biological behavior.

The biomaterials used in the bioink play a crucial role in providing mechanical support and creating microenvironments conducive to the cells within bioartificial organs. In an effort to optimize the microenvironment for the survival and functions of eHep cells, we formulated a hybrid bioink material named 3D-DLS gel in conjunction with gelatin and sodium alginate for the fabrication of 3DP-liver. Through systematic screening, we determined that a concentration of 5% for the 3D-DLS gel exhibited excellent 3D bioprinting performance. The LDCM, in conjunction with sacrificial gelatin, provided eHep cells with the necessary microenvironment and space for proliferation.

The achievement of mature liver functionality is a critical prerequisite for an artificial liver. In this study, we observed rapid proliferation and cluster formation of eHep cells within the 3DP-liver. The grid-like printing pattern facilitated ample oxygen and nutrient access for eHep cells. In comparison to 2D and organoid cultures, 3DP-liver exhibited up-regulated expression of liver function–related genes and demonstrated a closer resemblance to liver-specific capabilities, such as metabolic and detoxification functions, approaching levels observed in primary hepatocytes. This suggests that 3DP-liver constructed with 3D-DLS gel provides a more favorable culture environment for the proliferation and maturation of eHep cells, laying the foundation for 3DP-liver to exhibit mature liver functionality in vivo.

Ensuring an adequate number of functional hepatocytes is another pivotal factor influencing the therapeutic potential of 3DP-liver. Hepatocytes play a crucial role in most liver functions, including the metabolism of glucose, lipids, amino acids, and drugs. In human adults, the liver contains approximately3.6 × 10^11^ hepatocytes (*34*), while a mouse liver comprises around 1.5 × 10^8^ hepatocytes (*35*). In this study, we demonstrated that a 3DP-liver containing 2.7 × 10^7^ functional eHep cells, which accounts for approximately 18% of the normal mice liver cell count, could effectively compensate for the lost liver function in tyrosinemia or mice that underwent 90% hepatectomy. This highlights a promising heterotopic transplantation strategy using 3DP-liver for the treatment of end-stage liver failure. Compared to hepatocyte therapy, 3DP-liver transplantation presents a mix of advantages and disadvantages. The integration of transplanted hepatocytes into the recipient’s liver tissue poses a significant challenge. For patients with end-stage liver failure, such as those suffering from liver cirrhosis, the available space for integrating transplanted hepatocytes is often limited. 3DP-liver transplantation, however, effectively addresses these challenges by providing a sufficient mass of cells for therapy. Moreover, the 3DP-liver, being cross-linked with biomaterials, avoids the safety concerns related to the systemic diffusion of cells that are observed in hepatocyte transplantation. However, the use of biomaterials in transplantation also introduces stringent requirements for their biocompatibility.

Besides, constructing a large-scale 3DP-liver with a hepatic cell mass resembling that of the native human liver presents challenges in nutrient and oxygen exchange, necessitating vascularization (*36*). The timing and degree of vascularization are critical factors in determining when the 3DP-liver starts to function within the host body. For patients with chronic liver diseases, such as type I tyrosinemia and cirrhosis, the total loss of liver function occurs over a certain period. This gradual decline in liver function provides a window of opportunity for the transplanted 3DP-liver to establish vascular connections with the host’s body through the formation of new capillaries, thus beginning to perform liver functions and potentially saving the patient’s life. Conversely, acute liver failure, which may be triggered by conditions like hepatitis or the misuse of medication, leads to a rapid and complete loss of liver function. In such cases, OLT often represents the only viable treatment option. An artificial liver without a vascular system cannot immediately integrate with the host’s vascular network, which is a critical step for OLT success.

While considerable efforts have been directed toward establishing capillary networks in organoids, the incorporation of large blood vessels directly connected to the native blood vessel systems through surgical intervention is crucial. Hence, we successfully generated a 3DP-liver containing a blood vessel and effectively connected the artificial blood vessel to the native vessel system. This facilitated the gradual diffusion of biomolecules from artificial blood vessels while preventing blood leakage, demonstrating the feasibility of transplanting a large-scale bioartificial liver into native vascular systems. Nonetheless, OLT in rats presents its own set of challenges. While it is possible to connect the 3DP-liver to the rat’s vascular network using artificial blood vessels, the insufficient toughness of these vascular materials can result in blood leakage due to the rat’s active movements. There is a clear need for additional research to develop a stable system for the vascular preparation and fixation of the 3DP-liver within rats. This involves enhancing the durability of the vascular materials and refining the transplantation protocols. We are optimistic that with ongoing progress in medicine and biomedical engineering, the future will bring a broader selection of vascularization materials that are more suitable for transplantation.

## MATERIALS AND METHODS

### Isolation of mouse hepatocytes

Mouse hepatocytes were isolated from C57 mice aged 6 to 8 weeks. After preperfusion through the inferior vena cava, approximately 50 ml of calcium-free Earle’s balanced salt solution (EBSS) solution (Solarbio) was perfused into the liver at a rate of 5 to 7 ml/min. Once the blood in the liver disappeared, it was replaced with an EBSS solution containing 0.025% collagenase IV (Macklin), and perfusion continued at a volume of 100 to 150 ml. After perfusion, the liver tissue was placed in a culture dish and added with 10 ml of Dulbecco’s modified Eagle’s medium (DMEM) (Gibco). A pipette was used to gently agitate the interior of the liver repeatedly until the outflow solution from the hepatocytes became turbid. The hepatocyte suspension was subsequently filtered through a 100-μm sieve and centrifuged at 150*g* for 5 min to collect the cells. These cells were then resuspended in a Percoll (Sigma-Aldrich) solution (9 ml of Percoll + 1 ml of 10× EBSS with calcium and magnesium + 10 ml of DMEM) and subjected to centrifugation at 150*g* for 5 min to collect the cell precipitates. The precipitated cells were collected and washed twice with PBS. The purified hepatocytes were then ready for use in various experiments.

### Cell culture

After perfusion, mouse hepatocytes were seeded on type I collagen–coated dishes at a density of 1 × 10^4^ cells/cm^2^ in DMEM (Gibco) containing 10% fetal bovine serum (FBS) and 5% penicillin G and streptomycin (Gibco) for 6 hours. The culture medium was then switched to hepatocyte expansion culture medium: DMEM/F12 supplemented with ZnCl_2_, ZnSO_4_⋅7H_2_O (0.75 mg/liter), CuSO_4_⋅5H_2_O (0.2 mg/liter), MnSO_4_ (0.025 mg/liter), bovine serum ALB (2 g/liter), galactose (2 g/liter), ornithine (0.1 g/liter), proline (0.03 g/liter), nicotinamide (0.61 g/liter), insulin (10 mg/liter), transferrin (5.5 mg/liter), sodium selenite (6.7 μg/liter), transforming growth factor–α (50 ng/ml), epidermal growth factor (40 ng/ml), insulin-like growth factor II (100 ng/ml), hepatocyte growth factor (20 ng/ml), 1 μM sphingosine 1-phosphate, 5 μM forskolin, 10 μM dexamethasone, 10 μM Y-27632, 0.5 μM A-83-01, 3 μM CHIR99021, and 1% FBS (provided by Cellfarm, Shanghai Weien Biotechnology Co., Ltd.). HUVECs were cultured in endothelial cell medium (93% basal medium +5% FBS + 1% endothelial cell growth supplement + 1% penicillin/streptomycin solution) under 5% CO_2_ at 37°C.

### Material preparation

Liver tissues from C57 mice aged 6 to 8 weeks underwent isolation and perfusion with PBS. Subsequently, they were incubated in PBS containing 1% SDS (Biosharp) and 1% Triton X-100 (Diamond) for 72 hours for decellularization. The acellular liver tissue was freeze dried, and the resulting powder was dissolved in PBS containing 3% acetic acid and pepsin (1 mg/ml) (Sigma-Aldrich). This solution was then shaken at 37°C for 72 hours. NaOH was used to adjust the pH of the dissolved liver acellular matrix to neutral. After filtering out insoluble impurities using a mesh, the LDCM is stored at 4°C. Gelatin (Sigma-Aldrich) and sodium alginate (Solarbio) were dissolved in PBS, respectively, and stored at 4°C after sterilization. Before use, the liquid was heated to 37°C until it melted.

### 3D-bioprinted of 3DP-liver

A 3D mesh structure was printed using a biological 3D extrusion printer (SUNPBIOMAKER2i) from SUNP. The printer allows for the printing of grids with varying sizes, nozzle diameters, and print line distances. Bioink preparation involved mixing different concentrations of gelatin, sodium alginate, liver acellular matrix, and eHep cells (final concentration of 5 × 10^6^/ml) with HEM in specific proportions. The bioink was then placed at 4°C for 20 min. Typically, the bioink containing cells was printed in a 6-cm culture dish with an extrusion speed of 1 mm^3^/s. The printed liver structure was immersed in a 3% CaCl_2_ solution for rapid cross-linking, followed by extended culture in HEM.

### Rheology and mechanical behavior of 3D-DLS gel

The viscosity, storage modulus (*G*′), and loss modulus (*G*″) of the biological ink under different shear strains were measured using a rheometer (Anton Paar). The yield stress of the bioink was determined by applying stress-controlled deformation to the sample and measuring the resulting shear strain. The gel Young’s modulus of different species and concentrations was measured using a dynamic thermomechanical analyzer.

### Cell survival

Following printing, the 3DP-liver were cultured at 37°C with 5% CO_2_ for 3 days. The survival rate of eHep cells in bioprinting was assessed to evaluate the impact of the manufacturing and culture processes on cell activity. The cell survival rate was determined through a fluorescence live/dead test. Briefly, the 3DP-liver were washed with PBS and incubated with a mixture of calcein-AM (Beyotime) and PI (Beyotime) at 37°C for 30 min. Subsequently, the 3DP-liver were gently washed with PBS and observed under a fluorescence microscope. The cell survival rate was calculated by counting the number of cells [(live cells/total cells) × 100%] using ImageJ.

### Cell proliferation and loading efficiency

The eHep cells were transfected with a lentivirus carrying the overexpressed RFP, resulting in eHep-RFP cells, used for the fabrication of 3DP-liver. The proliferation of eHep cells within the 3DP-liver was monitored at days 0, 2, 4, and 6 using a fluorescence microscope. The changes in eHep-RFP fluorescence intensity were quantified using ImageJ. In addition, 3DP-liver cultured for 0, 2, 4, and 6 days were dissolved using a dissociation solution containing sodium citrate (55 mM), EDTA (20 mM), and NaCl (150 mM). The assessment of cell proliferation was determined by counting free eHep cells in the dissociation solution. The cell loading efficiency was calculated as [(number of cells at the bottom of the dish/number of cells in the bioprinting) × 100%].

### Quantitative polymerase chain reaction

Total RNA extraction from 3DP-liver and cells was carried out using Thermo Fisher Scientific’s TRizol. Following the manufacturer’s instructions, the HiScript III 1st Strand cDNA Synthesis Kit (Vazyme) was used to reverse transcribe 1 μg of RNA in a 20-μl reaction system. The resulting cDNA, after 50-fold dilution, underwent real-time quantitative polymerase chain reaction (PCR) on an ABI 7500 fast real-time PCR system (Applied Biosystems) using Taq Pro Universal SYBR qPCR Master Mix (Vazyme). Primer sequences are available upon request.

### Immunofluorescence

Bioprinted structures or cells were fixed in a 4% paraformaldehyde solution at room temperature for 30 min, followed by three washes with PBS. Before staining, the bioprinted structures or cells were blocked in PBS containing 0.1% Triton X-100 and 3% bovine serum ALB (Solarbio) for 30 min at room temperature. Subsequently, they were incubated overnight at 4°C with primary antibodies. After primary antibody incubation, the bioprinted structures or cells were washed three times with PBS and then incubated in the dark at 37°C for 2 hours with the appropriate fluorescently labeled secondary antibodies. Nuclear staining was performed using 4′,6-diamidino-2-phenylindole (Sigma-Aldrich). Primary antibodies included rabbit anti-ALB (Genetex), goat anti-Zo1 (Cell Signaling Technology), rabbit anti-Ki67 (Abcam) rabbit anti-fibronectin (Abcam), and mouse anti-FAH (Abcam).

### Validation of in vitro functions of eHep cells and 3DP-liver

In the ICG uptake assay, bioprinted structures or cells were cultured for 48 hours in a medium supplemented with 8-bromo-cAMP, pregnenolone-16α-carbonitrile, and progesterone, as per the manufacturer’s instructions. Subsequently, they were coincubated with ICG (1 mg/ml) at 37°C for 1 hour, washed three times with PBS, and then switched to HEM culture medium. The uptake/release of ICG was observed under a microscope. Bioprinted structures or cells were stained with ac-LDL (Yeasen) and periodic acid–Schiff (Solarbio), and the staining results were observed under a microscope. Different inducers were used to treat 3DP-liver and eHep cells for 48 hours, and the gene expression levels of *Cyp1a2* (3-methylcholanthracene, 10 μM, Sigma-Aldrich), *Cyp3a11* (rifampicin, 25 μM, Solarbio), *Cyp2e1* (rifampicin, 25 μM, Solarbio and ethyl alcohol; 0.5%, v/v), *Cyp3a41b* (3-methylcholanthracene, 10 μM, Sigma-Aldrich), and *Cyp1a1* (rifampicin, 25 μM, Solarbio and barbituric acid, 10 μM, Aladdin) were detected. The P450-Glo *Cyp1a2* Induction/Inhibition Assay (Promega) was used, following the manufacturer’s instructions, to detect *Cyp1a2* enzyme activity in different states.

### RNA sequencing data processing

RNA extraction from matched samples intended for DNA methylation analysis was accomplished using RNeasy Kits (Qiagen, catalog number 75144). Subsequently, deoxyribonuclease I treatment was administered to the extracted RNA (Thermo Fisher Scientific, catalog number AM2222), following established protocols. RNA sequencing was performed at Genesky using the TruSeq Stranded mRNA Library Preparation (Genesky) method. In summary, intact RNA underwent fragmentation, end repair, adapter ligation, and PCR amplification following the Illumina protocol. The resulting libraries were then sequenced on the Illumina HiSeq 2000 platform. After rigorous quality control, STAR was used to process sequence data and generate read alignments against the hg19 reference genome. Raw read counts for annotated genes were obtained using feature Counts with default settings, and subsequent normalization and analysis were conducted using DESeq2. Validation of the RNA sequencing data was achieved through real-time PCR. Genes were considered statistically different when the *P* value was <0.05 and the fold change was >1.2.

### Animal experiments

For the transplantation of 3DP-liver into the mesenteric region of C57BL/6NCrl mice, survival duration was meticulously recorded. One week after transplantation, the intravenous injection of FID (Sigma-Aldrich) was used to observe the connectivity between mice and the 3DP-liver, orbital venous blood samples were collected for white blood cell, red blood cell, hemoglobin, mean platelet volume, mean corpuscular hemoglobin, hematocrit, and mean corpuscular hemoglobin concentration analysis.

*Fah^−/−^* mice were maintained with NTBC (7.5 mg/liter) in the drinking water. One week after transplanting 3DP-liver into the mesenteric region of *Fah^−/−^* mice, NTBC was withdrawn from the drinking water. A control group consisting of eight *Fah^−/−^* mice without any transplantation also had NTBC withdrawn. Survival duration and changes in body weight were documented. At 3 weeks after NTBC withdrawal, blood samples were collected via retro-orbital sinus sampling. The collected blood was allowed to stand at 4°C for 1 hour, followed by centrifugation at 10,000*g* for 20 min. The supernatant was used for the determination of ALT, AST, ALB, TBA, TBIL, DBIL, ALP, and amino acid levels. At 3 weeks after NTBC withdrawal, livers were collected from both control and experimental groups of mice and subjected to H&E staining.

One week after transplanting 3DP-liver into the mesenteric region of C57 mice, a 90% hepatectomy was performed. Following the surgery, daily intraperitoneal injections of glucose were administered to both control and experimental groups of mice. Survival duration was recorded. At 36 hours after hepatectomy, mouse serum was collected in the same manner to detect the levels of ALT, AST, ALB, TBA, TBIL, DBIL, and ALP. All animal experiments were approved by the animal ethics committee (IRM-DWLL-2022179, no. 2022-08).

### Vessel imaging

eHep-RFP–engineered 3DP-liver was transplanted into the mesenteric region of Tek-Cre, Ai47-GFP mice. Seven days later, the bioprinting was extracted, and the alignment status between the 3DP-liver and the blood vessels within the mouse was observed under a stereo microscope (Olympus).

### Preparation of 3D-printed livers with artificial blood vessels

We assembled a coaxial extrusion system by combining two commercial needles (8G and 13G) and connecting them with epoxy resin. This system, compatible with the SUNP BIOMAKER 2i bioprinter, executed predesigned vascular bioprinting protocols. HUVECs at a final concentration of 5 × 10^6^/ml were suspended in a premade bioink mixture comprising 8% GelMA (SUNP), 1% sodium alginate, 0.25% LAP (Sigma-Aldrich), and 1.6% PEO (Sigma-Aldrich). The bioprinting sequence involved the lower part of 3D-printed livers, blood vessels, and the upper part of 3D-printed livers. Blood vessels rapidly cross-linked and solidified within the 3D-printed livers under the influence of 405-nm ultraviolet light and 3% CaCl_2_, embedding them into the printed liver. Long-term cultivation was subsequently performed using HEM+ECM culture medium.

### Diffusion of Cy7 fluorescent dye and glucose accumulation in 3DP-liver with artificial blood vessels

In vitro perfusion of the vascularized 3DP-liver was conducted using PBS containing Cy7 fluorescent dye (0.1 mg/ml, APExBIO). The perfusion flow rate was adjusted to 5 to 7 ml/min. Fluorescence intensity at different regions of the 3DP-liver was monitored at various time points, specifically at 0, 10, 15, 30, 60, and 120 min, after the start of perfusion using the Maestro imaging system (CRi Maestro). Quantitative analysis of fluorescence intensity was performed using ImageJ. To validate the permeability of blood vessels to glucose, the blood vessels were placed in a 6-cm dish containing PBS. The ends of blood vessels were positioned outside the dish. In vitro glucose (100 mg/dl) perfusion of the vascular specimen was conducted, and glucose concentration within the PBS of the culture dish was measured at different time points following the start of perfusion using a glucose detection kit (Solarbio).

### Transplantation of 3DP-liver with artificial blood vessels

A classical in situ transplantation experiment of the vascularized 3DP-liver was conducted using 10- to 12-week-old Sprague-Dawley rats. Rats were rapidly anesthetized using 3% isoflurane before surgery. Hemostatic clamps were used to temporarily occlude the distal ends of the inferior vena cava and portal vein to halt blood flow during the procedure. Cuffs (polyimide tubes) were placed at both ends of the inferior vena cava and portal vein and secured with 6-0 sutures. The two ends of the vascularized 3DP-liver were then inserted into the cuffs, and the hemostatic clamps were removed to establish blood perfusion.

### Statistical analysis

The results were expressed as the mean ± SD. Differences were assessed for statistical significance through Student’s *t* test or analysis of variance (ANOVA) analysis, with a significance threshold set at a *P* value less than 0.05.
