# Supplementary material for: Revitalizing liver function in mice with liver failure through transplantation of 3D-bioprinted liver with expanded primary hepatocytes
Source: Sci Adv. 2024 Jun 7;10(23):eado1550. doi: 10.1126/sciadv.ado1550 (PMC11160470; doi:10.1126/sciadv.ado1550)
Supplement: Supplementary file 1 — Figs. S1 to S29 Legends for movies S1 and S2 [file sciadv.ado1550_sm.pdf]

Science Advances
AAAS

Supplementary Materials for

Revitalizing liver function in liver failure mice through transplantation of 3D-bioprinted liver with expanded primary hepatocytes

Bo Deng *et al.*

Corresponding author: Pengyu Huang, huangpengyu@yeah.net; Lanxia Liu, liulanxiabme@163.com; Huayu Yang, dolphinyahy@hotmail.com

Sci. Adv. **10**, eado1550 (2024)
DOI: 10.1126/sciadv.ado1550

The PDF file includes:

Figs. S1 to S29
Legends for movies S1 and S2

Other Supplementary Material for this manuscript includes the following:

Movies S1 and S2

Fig. S1.

hematoxylin and eosin (H&E) staining of the mice liver after decellularization. Scale bar: 100μm.

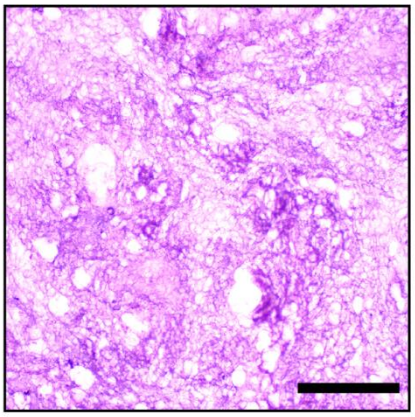

Fig. S2.

Representative images of 10% LDCM at 37°C/4°C.

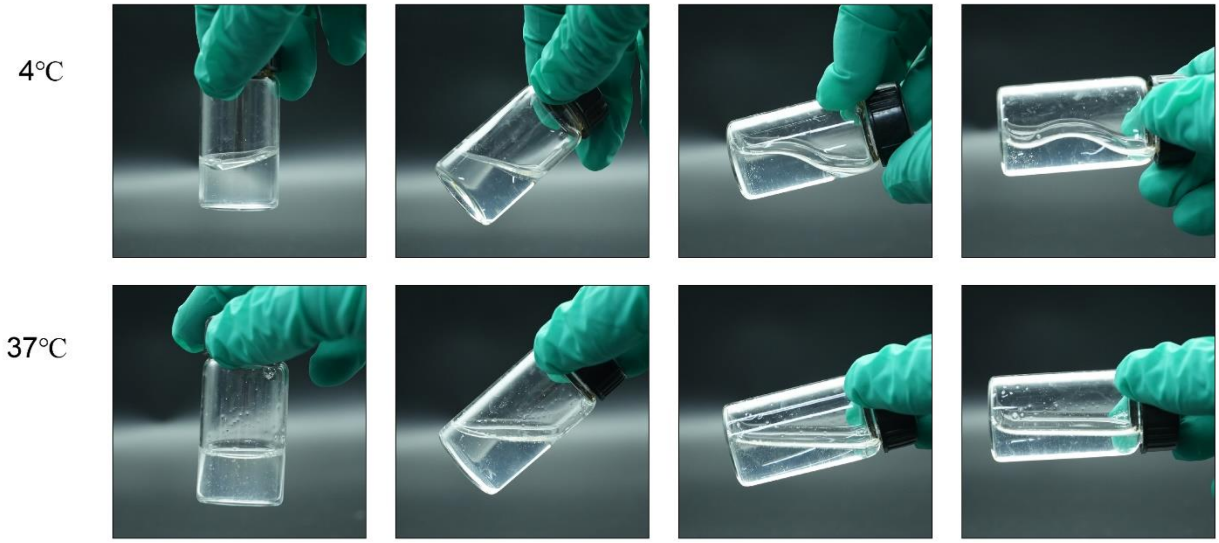

Fig. S3.

Proliferation of eHep-RFP cells analyzed in the 3D-DLS gel (5%) and the gelatin&sodium alginate gel.

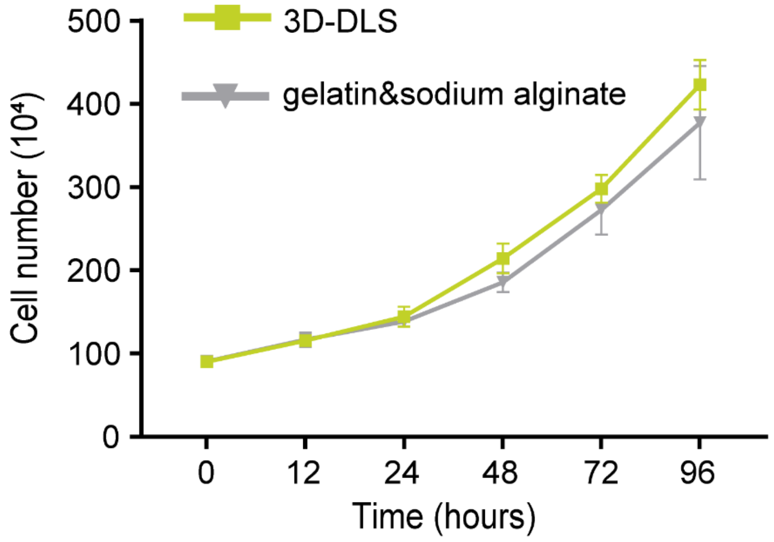

Fig. S4.

Hepatic marker gene expressions of hepatocytes in the gelatin&sodium alginate gel-based 3D culture (gelatin&sodium alginate) and the 3DP-liver (3D-DLS) after 5 days of *in vitro* culture. *P<0.05.

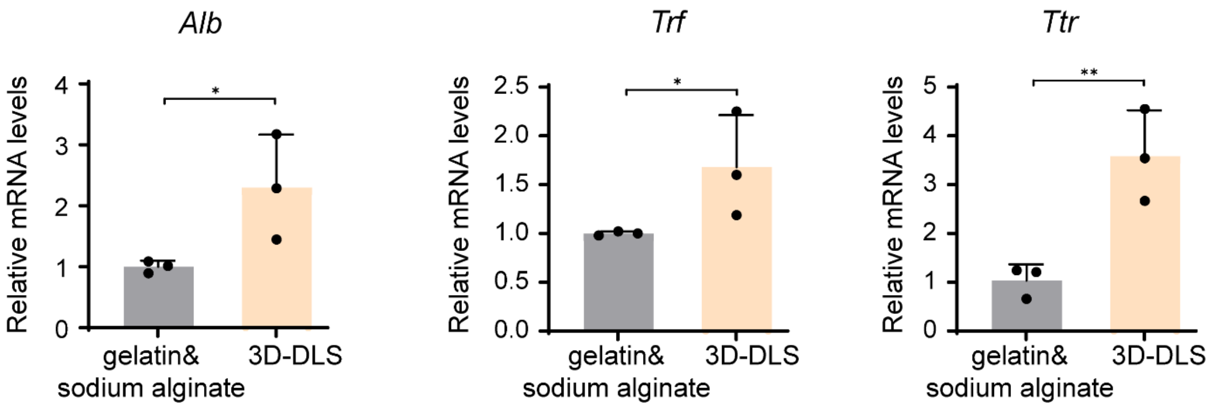

Fig. S5.

qPCR analysis of the expression of the *Prkx* and *Prkaca* in 2D-culture (2D), Matrigel-based 3D culture (matrigel), freshly isolated mice primary hepatocytes (PH), and 3DP-liver (3D) after 5 days of *in vitro* culturing. *P<0.05. **P<0.01. ***P<0.001.

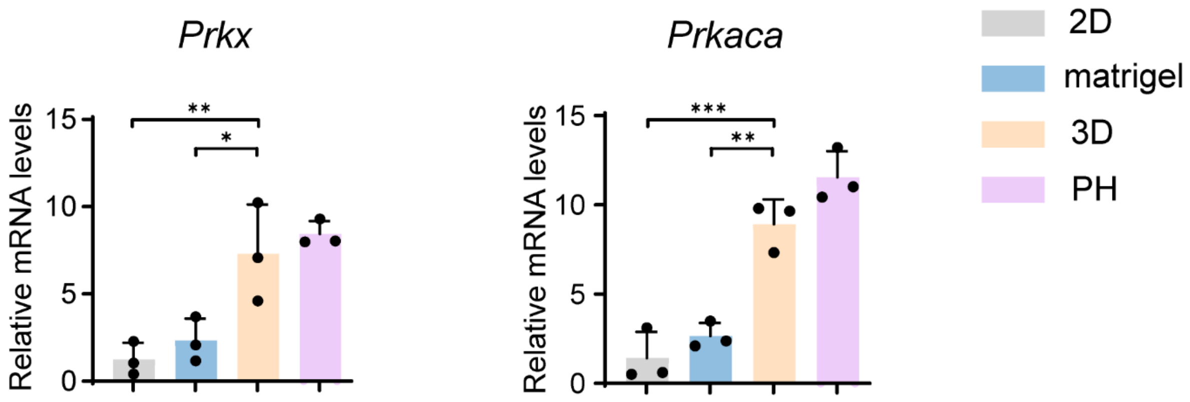

Fig. S6.

GSEA of mRNA profiling results (cAMP signaling pathway) from 2D-cultured eHep cells and 3DP-liver. 2D, 2D-cultured eHep cells; 3D, 3DP-liver.

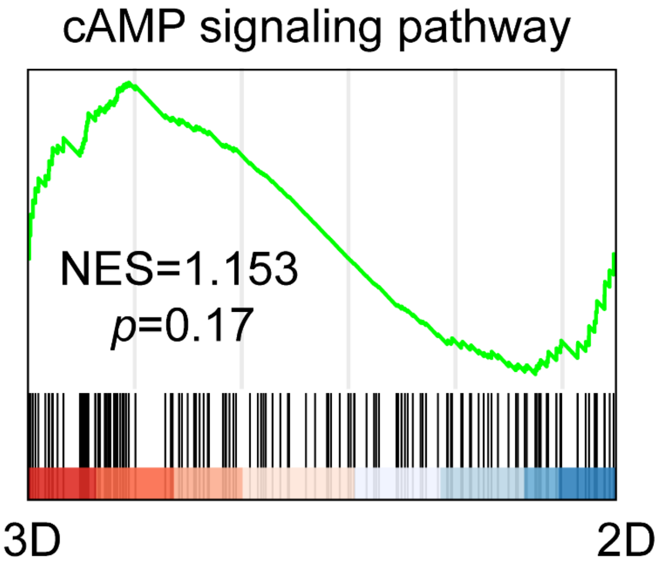

Fig. S7.

PCA for RNA-seq. Pri, primary hepatocytes; 2D, 2D-cultured eHep cells; 3D, 3DP-liver.

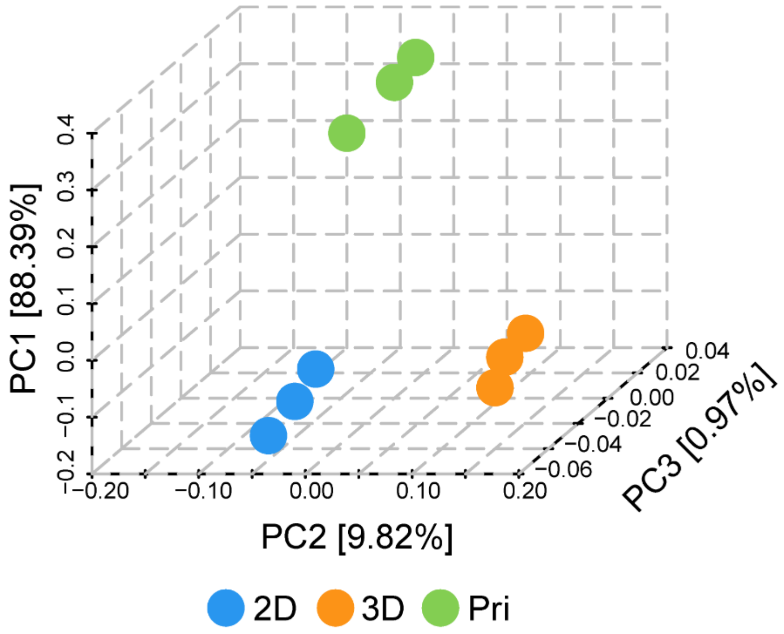

Fig. S8.

Venn diagram showing differentially expressed genes (DEGs) of pairwise comparisons. Purple, genes downregulated in 3D compared with Pri; green, genes downregulated in 3D with 2D; yellow, genes upregulated in 3D compared with Pri; red, genes upregulated in 3D with 2D. Number of DEGs are indicated in the diagram. Pri, primary hepatocytes; 2D, 2D-cultured eHep cells; 3D, 3DP-liver.

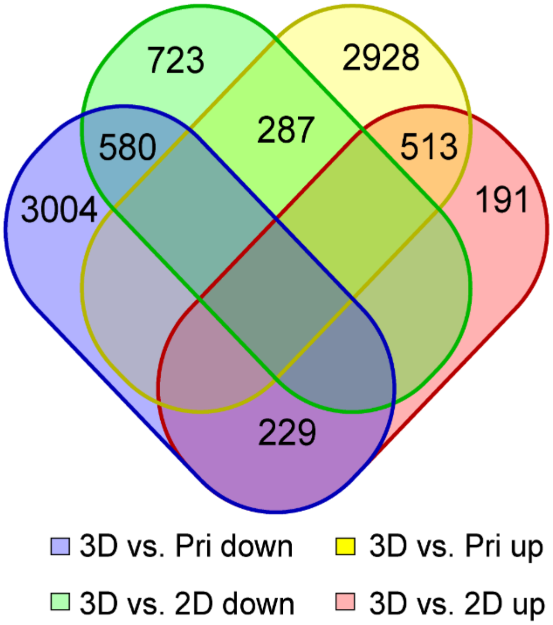

Fig. S9.

Volcano plots of differential gene expressions between 2D-cultured eHep cells and 3DP-liver. 2D, 2D-cultured eHep cells; 3D, 3DP-liver.

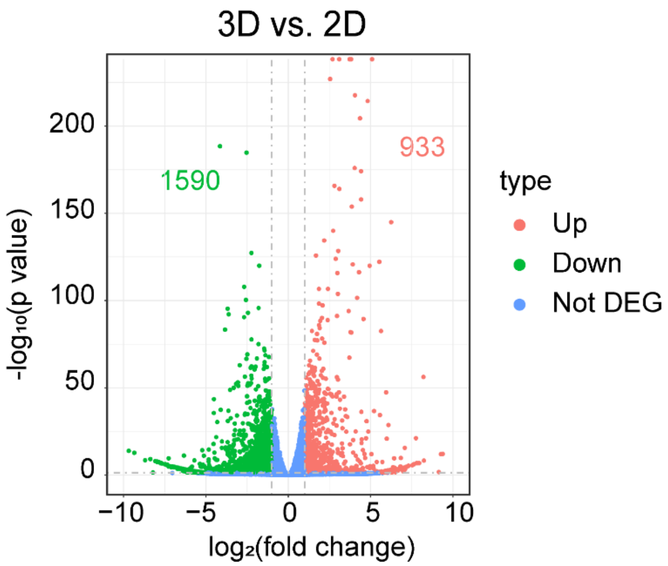

Fig. S10.

GSEA of mRNA profiling results from 2D-cultured eHep cells and 3DP-liver. 2D, 2D-cultured eHep cells; 3D, 3DP-liver. Pri, primary hepatocytes.

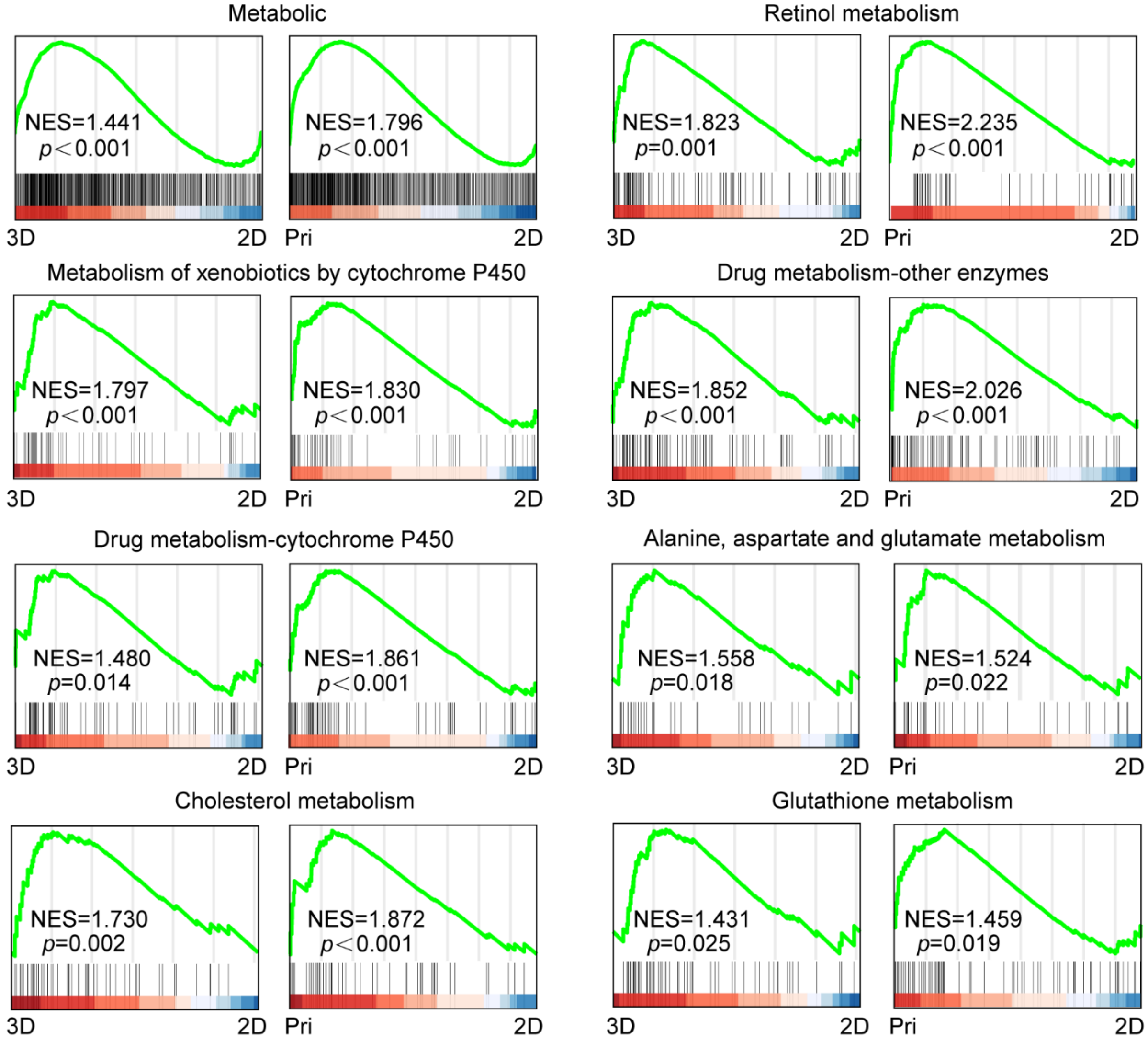

Fig. S11.

mRNA levels of uninduced *Cyp3a41b* and induced *Cyp3a41b* determined by qPCR. *Cyp3a41b* was induced by 3-methylcholanthrene. TTF, Tail-tip fibroblasts; 2D, 2D-cultured; matrigel, Matrigel-based 3D culture; 3D, 3DP-liver; ***P<0.001.

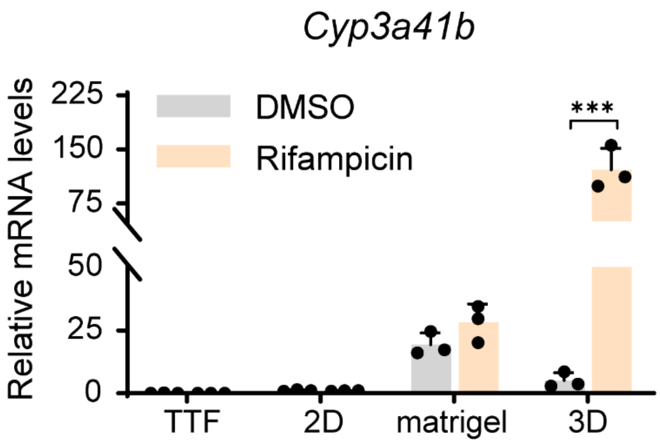

Fig. S12.
mRNA levels of uninduced *Cyp1a1* and induced *Cyp1a1* determined by qPCR. *Cyp1a1* was induced by rifampicin and barbituric acid. TTF, Tail-tip fibroblasts; 2D, 2D-cultured; matrigel, Matrigel-based 3D culture; 3D, 3DP-liver; ***P<0.001.

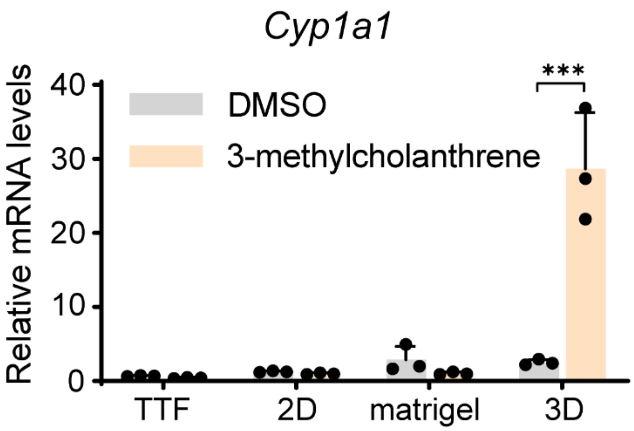

Fig. S13.
Heatmap showing upregulation genes related to the cell adhesion molecules pathway in 3DP-liver. 2D, 2D-cultured eHep cells; 3D, 3DP-liver.

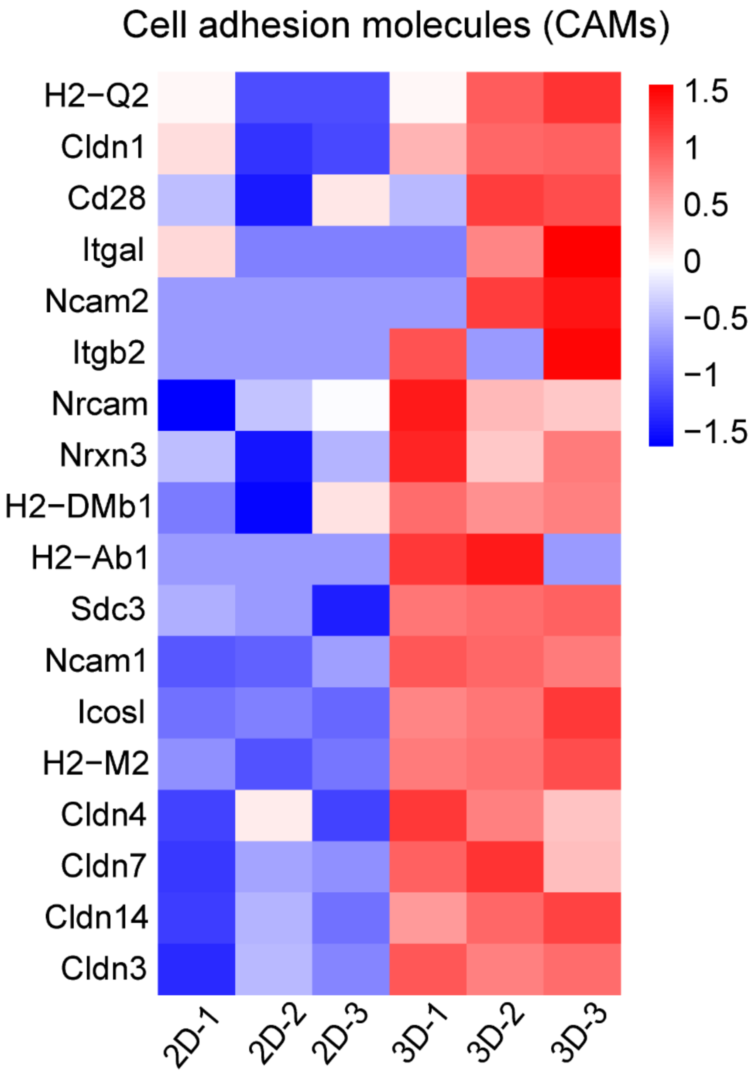

Fig. S14.
Heatmap showing upregulation genes related to the Extracellular Matrix (ECM)-receptor pathway in 3DP-liver. 2D, 2D-cultured eHep cells; 3D, 3DP-liver.

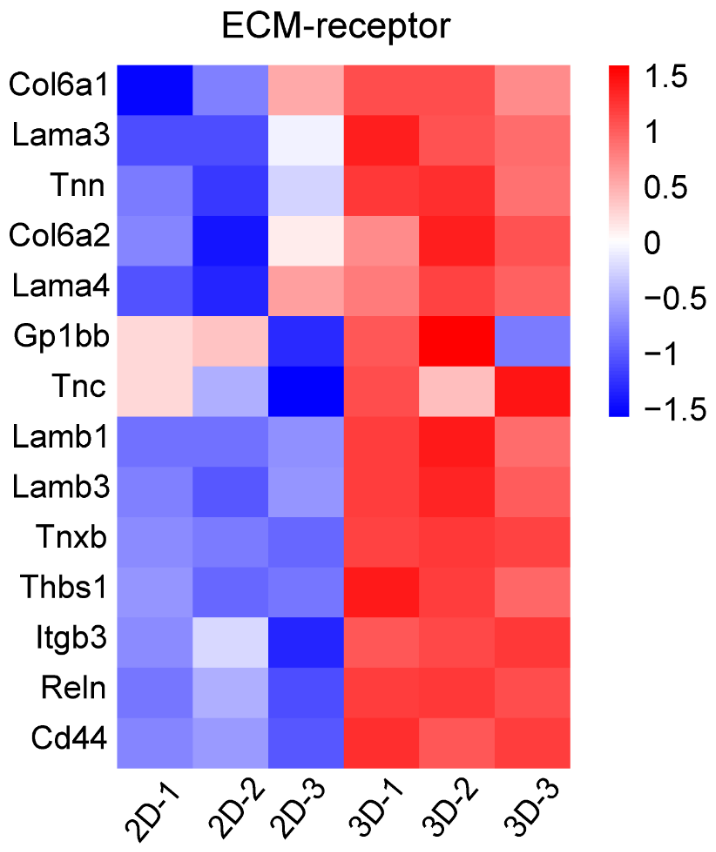

Fig. S15.
Representative images of the 3DP-liver transplanted into mice after 7 days and after 1 month. Scale bar: 5mm.

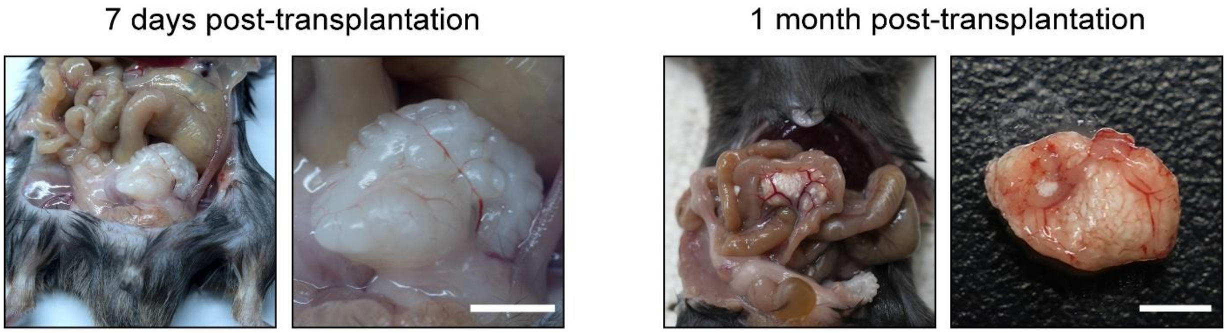

Fig. S16.
eHep cells loading efficiency of 3DP-liver during *in vitro* cultivation (n=3).

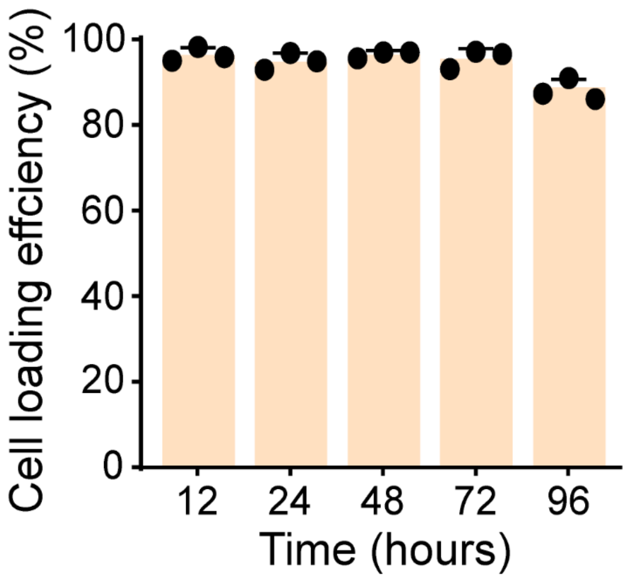

Fig. S17.
Survival curve of 3DP-liver-transplanted C57BL/6NCrl mice (n=8).

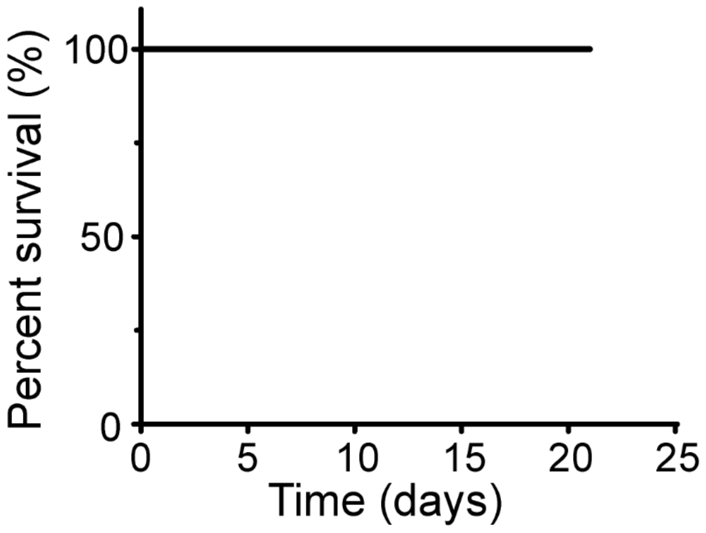

Fig. S18.

hematological toxicity analysis of 3DP-liver after transplantation into mice for 7 days. WT, Wild type; transplant, mice transplanted with 3DP-liver; WBC, white blood cell; RBC, red blood cell; HGB, hemoglobin; MPV, mean platelet volume; MCH, mean corpuscular hemoglobin; HCT, hematocrit; MCHC, mean corpuscular hemoglobin concentration. ns not significant.

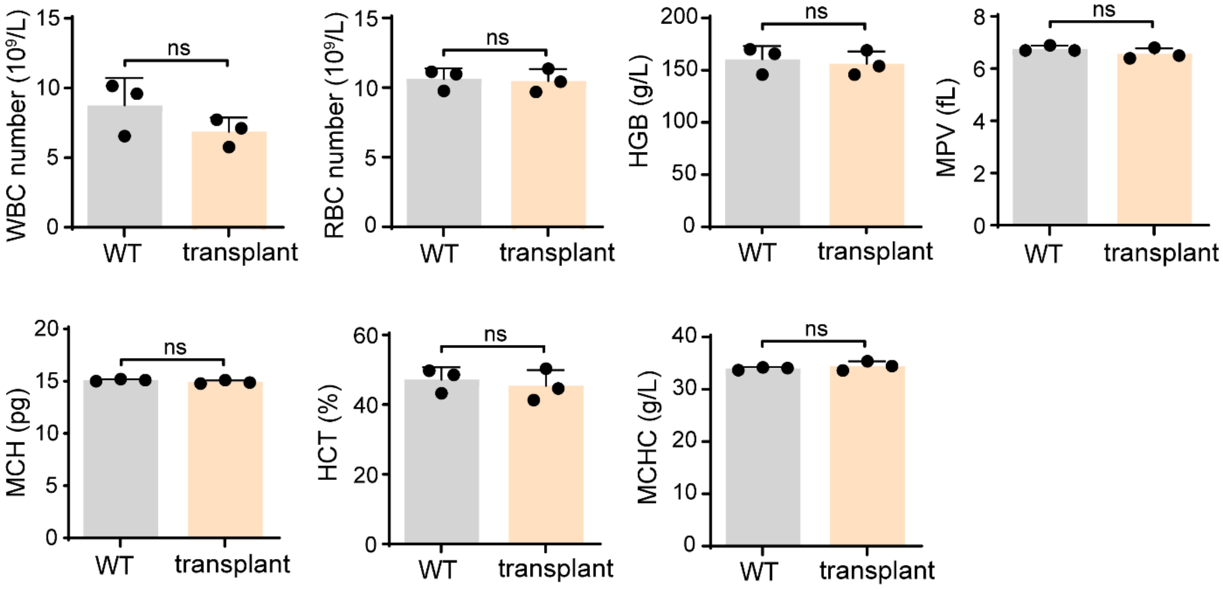

Fig. S19.
Kinetics of 3DP-liver vascularization after transplantation into the mesentery of Tek-Cre, Ai47-GFP mice after 1, 3, and 7 days. Scale bar: 5mm.

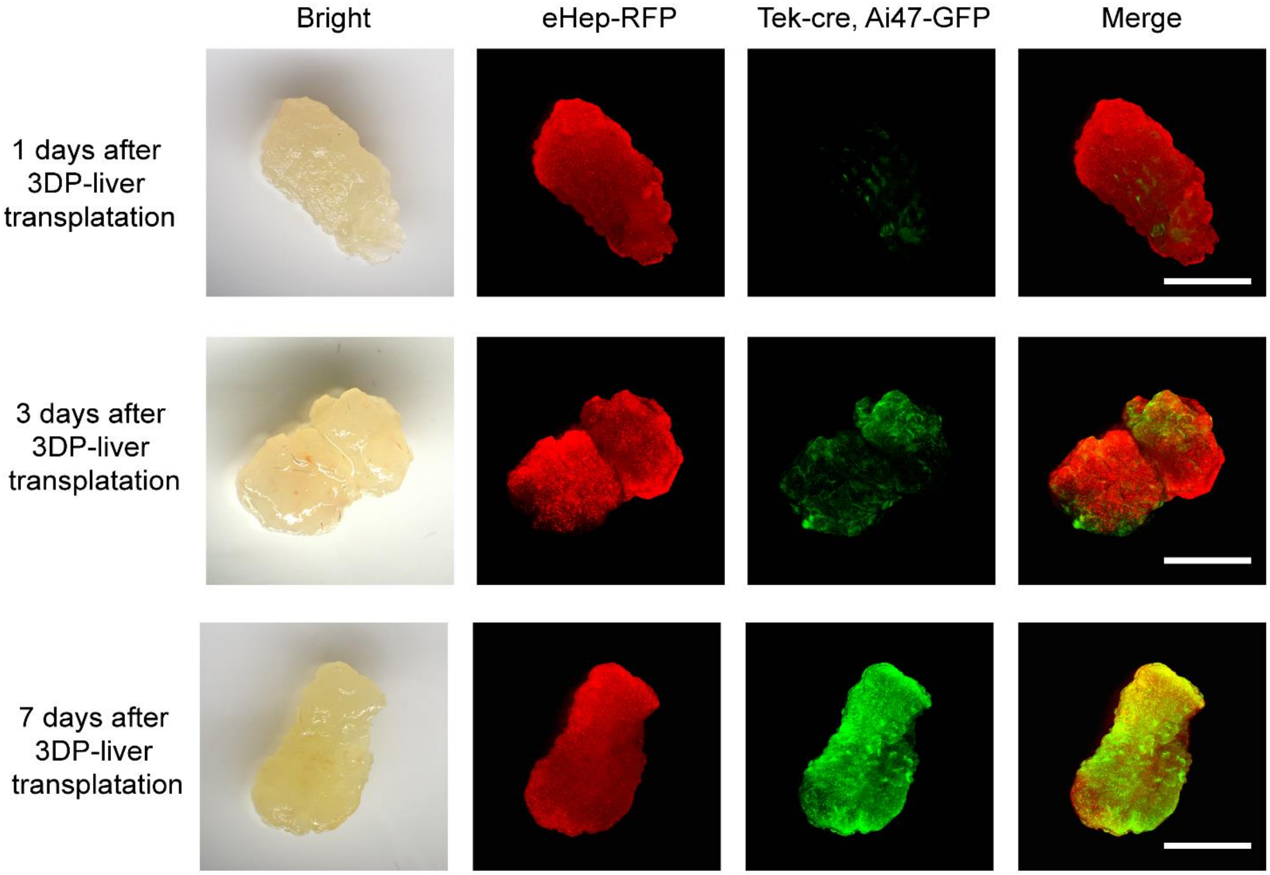

Fig. S20.
Mean neovascularization density of the 3DP-liver after transplantation into the mesentery of Tek-Cre, Ai47-GFP mice.

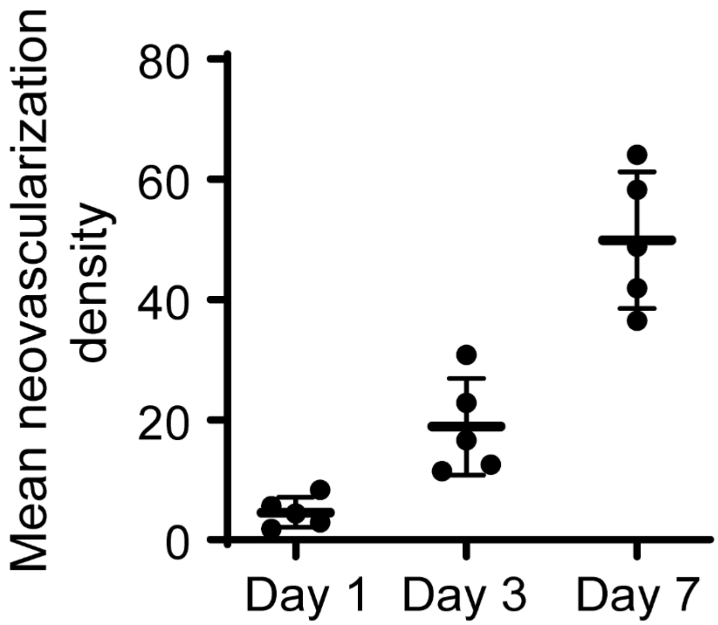

Fig. S21.
Vascularization of the 3DP-liver (7%) after transplantation into the mesentery of Tek-Cre, Ai47-GFP mice on day 3. Scale bar: 5mm.

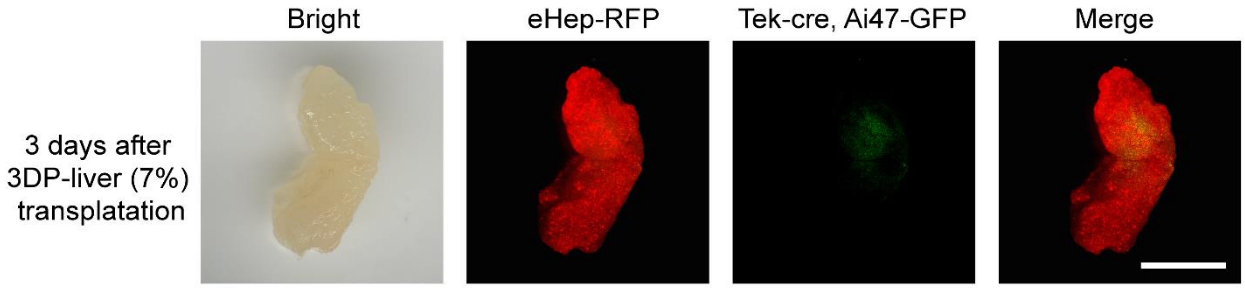

Fig. S22.
Immunofluorescent staining of Fibronectin on 3DP-liver sections after transplantation. Scale bar: 100μm.

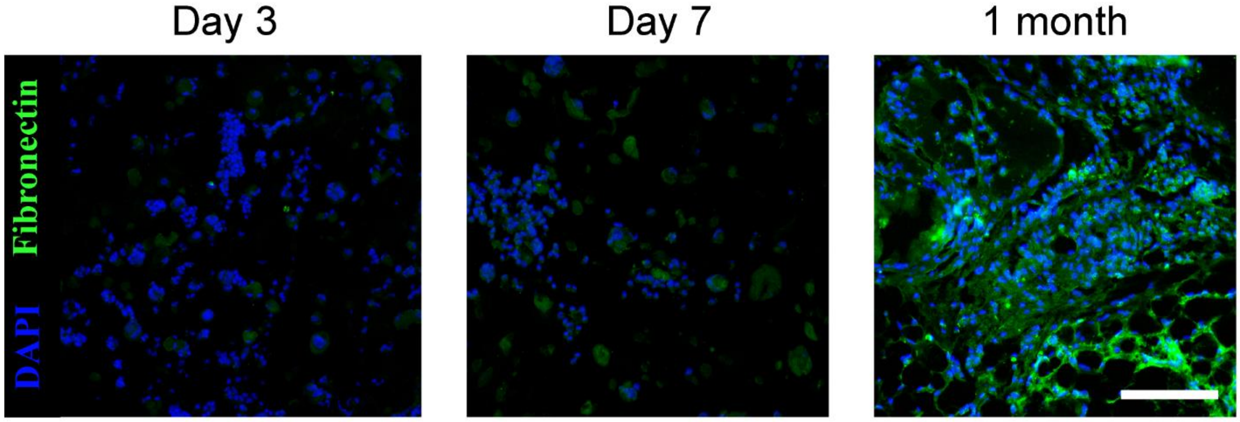

Fig. S23.
eHep cell numbers of the 3DP-liver at different timepoints after 3DP-liver transplantation.

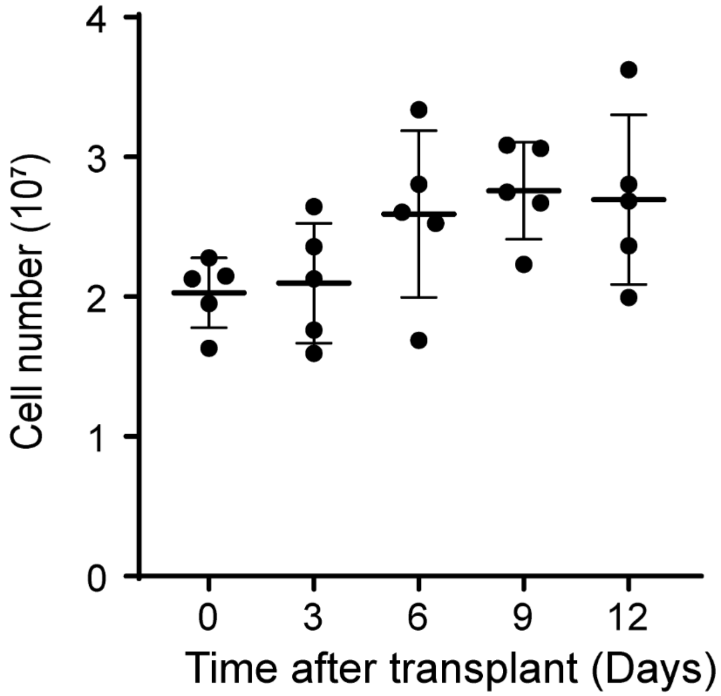

Fig. S24.
Proliferation of eHep cells in 3DP-livers analyzed by Ki67 immunofluorescent staining 1 month after transplantation. Scale bar: 100µm.

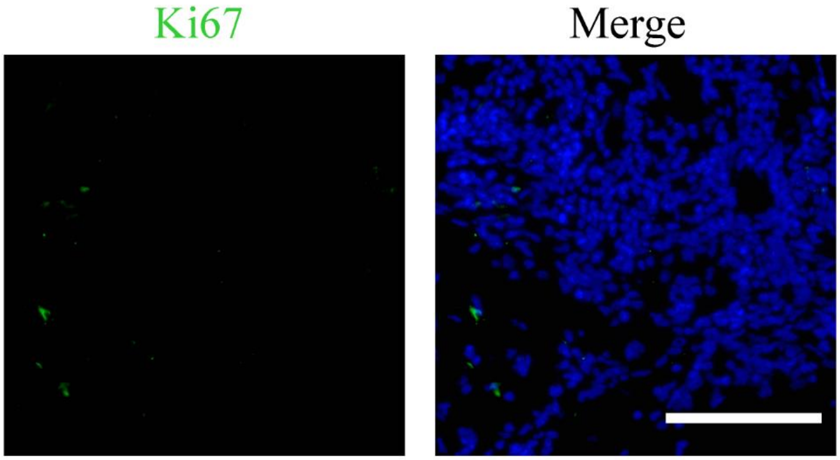

Fig. S25.
Serum levels of the indicated amino acids in wildtype (WT, n=3), *Fah*$^{-/-}$ mice (*Fah*$^{-/-}$, n=3), and 3DP-liver-transplanted *Fah*$^{-/-}$ mice (3DP-liver, n=3, sera collected at 3 weeks after transplantation). *P<0.05. **P<0.01. ***P<0.001.

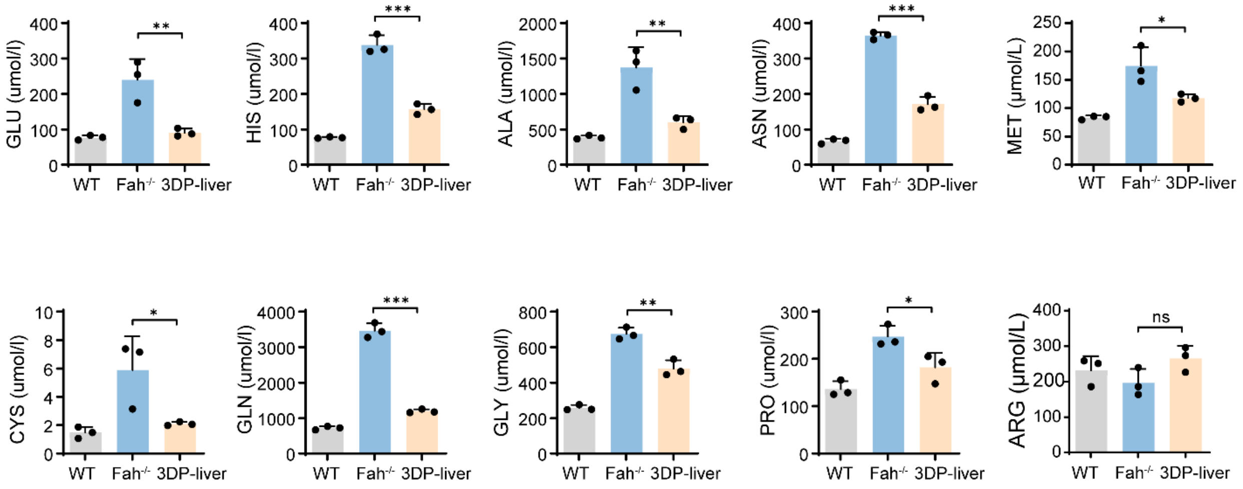

Fig. S26.
Representative images of liver from wildtype, *Fah*$^{-/-}$ mice, and *Fah*$^{-/-}$ mice with 3DP-liver transplanted. Scale bar: 1cm.

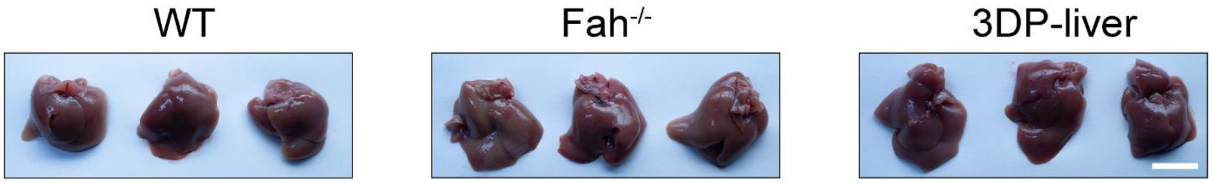

Fig. S27.
Survival curve of 3DP-liver-transplanted 90% hepatectomy mice (3DP-liver, n=10) and control 90% hepatectomy mice (sham-operated, n=10).

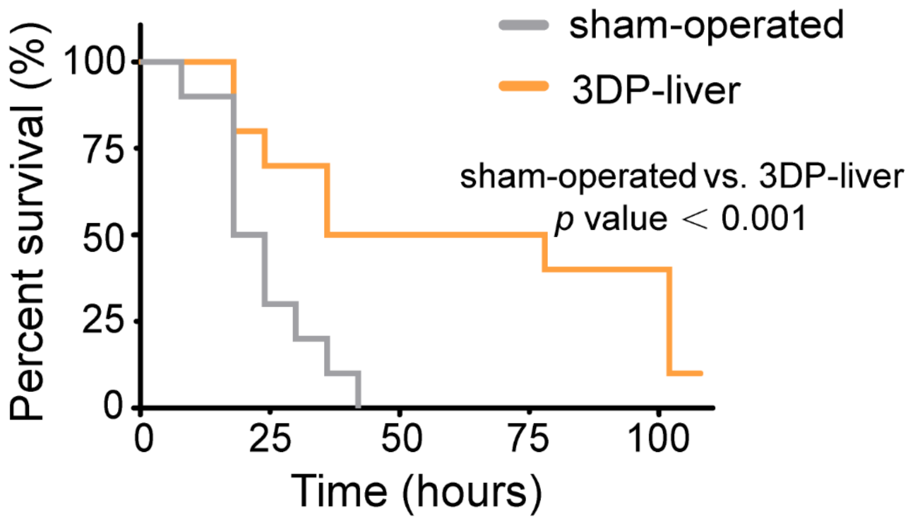

Fig. S28.

Serum levels of ALT, AST, ALB, TBA, TBIL, DBIL and ALP in wildtype (WT, n=3), 90% hepatectomy mice (sham, n=3), and 3DP-liver-transplanted 90% hepatectomy mice (3DP-liver, n=3, sera collected at 2 days after transplantation). ns not significant. **P<0.01. ***P<0.001.

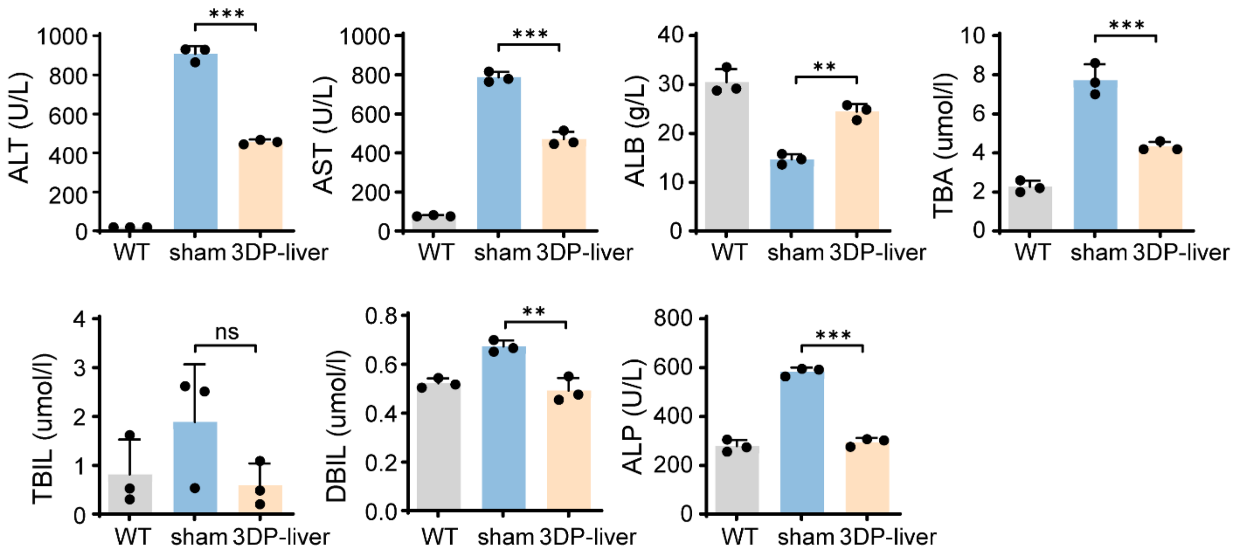

Fig. S29.
SEM images of the artificial blood vessel wall. Scale bar: 50µm.

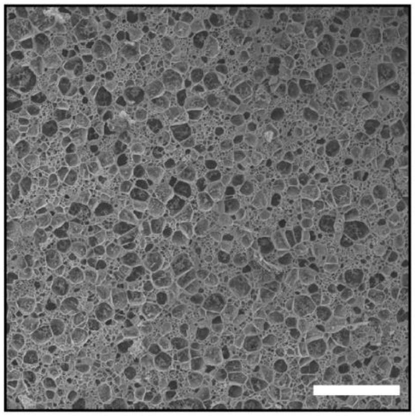

Movie S1.
Surgical anastomosis of the 3DP-liver with artificial blood vessels in rat. Blood perfusion

Movie S2.
Surgical anastomosis of the 3DP-liver with artificial blood vessels in rat. Blood circulation of rat
